# Evaluating causes of error in landmark-based data collection using scanners

**DOI:** 10.1371/journal.pone.0187452

**Published:** 2017-11-03

**Authors:** Brian M. Shearer, Siobhán B. Cooke, Lauren B. Halenar, Samantha L. Reber, Jeannette E. Plummer, Eric Delson, Melissa Tallman

**Affiliations:** 1 Ph.D. Program in Anthropology, The Graduate Center (CUNY), New York, New York, United States of America; 2 New York Consortium in Evolutionary Primatology, New York, New York, United States of America; 3 NYCEP Morphometrics Group, New York, New York, United States of America; 4 Center for Functional Anatomy and Evolution, Johns Hopkins University School of Medicine, Baltimore, Maryland, United States of America; 5 Department of Biology, Farmingdale State College (SUNY), Farmingdale, New York, United States of America; 6 School of Forensic and Applied Sciences, University of Central Lancashire, Preston, United Kingdom; 7 Department of Archaeology, University of Sheffield, South Yorkshire, United Kingdom; 8 Division of Vertebrate Paleontology, American Museum of Natural History, New York, New York, United States of America; 9 Department of Anthropology, Lehman College (CUNY), Bronx, New York, United States of America; 10 Department of Biomedical Sciences, Grand Valley State University, Grand Valley, Michigan, United States of America; Max Planck Institute for Evolutionary Anthropology, GERMANY

## Abstract

In this study, we assess the precision, accuracy, and repeatability of craniodental landmarks (Types I, II, and III, plus curves of semilandmarks) on a single macaque cranium digitally reconstructed with three different surface scanners and a microCT scanner. Nine researchers with varying degrees of osteological and geometric morphometric knowledge landmarked ten iterations of each scan (40 total) to test the effects of scan quality, researcher experience, and landmark type on levels of intra- and interobserver error. Two researchers additionally landmarked ten specimens from seven different macaque species using the same landmark protocol to test the effects of the previously listed variables relative to species-level morphological differences (i.e., observer variance versus real biological variance). Error rates within and among researchers by scan type were calculated to determine whether or not data collected by different individuals or on different digitally rendered crania are consistent enough to be used in a single dataset. Results indicate that scan type does not impact rate of intra- or interobserver error. Interobserver error is far greater than intraobserver error among all individuals, and is similar in variance to that found among different macaque species. Additionally, experience with osteology and morphometrics both positively contribute to precision in multiple landmarking sessions, even where less experienced researchers have been trained in point acquisition. Individual training increases precision (although not necessarily accuracy), and is highly recommended in any situation where multiple researchers will be collecting data for a single project.

## Introduction

Over the last decade, landmark based three-dimensional geometric morphometrics (3DGM) utilizing digital specimen scans has become an increasingly integral tool in the fields of physical anthropology and paleontology. 3DGM allows researchers to analyze complex (i.e., non-linear) shape data through the application of landmarks to anatomically homologous points on multiple specimens [1]. Landmarks can be acquired either directly from a physical specimen, as with a Microscribe digitizer, or digitally via a computer program, such as Landmark Editor [2], on a virtual rendition of a bone. The latter method has become popular recently with the decreased price and increased ease-of-use of surface scanners, which allow researchers to create a permanent digital copy of a specimen for later use in landmark-based analyses and/or for storage and sharing with other researchers via an online database (e.g., www.morphosource.org). Many researchers have also begun using computed tomography scanners (CT) to digitally render their specimens when interested in both internal and external morphology, as dramatic increases in processing power of commercial computers and greater access to CT scanners has made this technology more practical in non-medical research (see [3,4,5] for reviews). Digital renderings of bony tissue from both surface and CT scanners are often treated as equivalent by researchers (e.g., [6]) and are used interchangeably based upon availability. However, there is no broadly consistent protocol for rendering digital scans or for applying landmarks to digital models, and the possibility that landmark-based 3DGM studies can potentially suffer from problems of inter- and intraobserver error as a result of these variables has not been thoroughly investigated (but see [7]).

In any landmark-based study using digitally rendered specimens there are multiple factors which may introduce error. Technological sources of error potentially include scanner type and brand (which inherently vary in their surface capture abilities based on design features) resolution at which a specimen is scanned, and the fitting and smoothing algorithms that may be used in post-processing of the surfaces that may differ per proprietary software programming idiosyncrasies. Scanning protocol-based sources of error result from the individual choices made by a researcher regardless of what scan technology they choose to utilize, and may include scanning methods (e.g., particular number of frames, scanning angle, or overall number of image families used at the discretion of the researcher), or reconstruction/rendering methods used that may include differences in a particular scan model refinement method (e.g., to what extent the “Mesh Doctor” function in Geomagic Studio or Wrap is used rather than a targeted refinement protocol using other available tools). User-based sources of error include differences in data collection experience among researchers, inherent researcher tendencies for precision and accuracy, and comprehension of instructions. Data collection-based sources of error involve repeatability of landmark protocols.

Landmarks are traditionally classified into three different types based on potential for anatomical homology. Type I landmarks are generally the most desirable type of landmark because of their ease of reproducibility and in identification of anatomical homology. They can be defined as points where multiple tissues intersect [8], for example, where the coronal and sagittal sutures meet (Bregm(A). Type II landmarks can be defined as points of potential homology that are based only on geometric evidence. Type II landmarks are often placed on the maxima or minima of structures, such as the tip of the canine. Type III landmarks are mathematically deficient in at least one coordinate, and are generally defined only with respect to other landmarks in that they characterize more than a single region of an object’s form [8]. Landmark types II and III are less desirable than Type I, as they are more difficult to accurately find and precisely mark, and generally describe structures that are not necessarily homologous in the traditional sense of the word [8], but are more likely to be mathematically or geometrically homologous. More recent research has introduced semilandmarks from 2D morphometrics [9,10] to 3DGM studies (e.g., [11]). Semilandmarks are used to compare the shapes of biological curves that are suspected to hold some functional or phylogenetic information but present an even more difficult case of repeatability. These curves are usually anchored with anatomically homologous landmarks which are also spaced equidistantly between the anchoring points. These points are then “slid” into their most “homologous” positions prior to multivariate analyses by minimizing either the bending energy or Procrustes distances in the sample (see [12] for an example of how both of these methods affect data processing). Semilandmark curves have been demonstrated to be most useful when applied over large surfaces that do not contain numerous traditional landmarks (e.g., the occipital bone of the cranium [13] or the trochlear surface of the tibia [14]).

Several researchers have conducted small-scale error studies examining between-scanner error and interobserver error with non-GM data and their results mostly suggest these types of error are of minimal concern. For example, Tocheri et al. [15] conducted an error study using non-landmark-based methods, in which they examined the variance in surface shape metrics of gorilla tarsals as collected by two researchers on virtual 3D models generated from both CT and laser surface scanners. They found that laser scan surfaces and those extracted from CT scans were not distinguishable, and that the two individuals who rendered and collected the data did not do so in a statistically different fashion. Likewise, Sholts et al. [16] measured scan model area and volume when constructed with multiple protocols and by two different individuals. They report intra- and interobserver error in scan construction at 0.2% and 2% variance, respectively, which they interpret as non-significant for scan sharing.

In a study conceived concurrently with this one, Robinson and Terhune [17] compared both inter- and intraobserver error rates between the two researchers on 14 differently sized crania of 11 primate taxa using traditional linear measurements, tactile 3D landmarking (i.e., Microscribe), and digital landmarking of computer rendered models. In regards to variance levels when applying landmarks to digital 3D models for morphometric analyses, they demonstrate negligible differences in rates of error between how scans were created (e.g., NextEngine vs CT), and that interobserver variation is higher than both intraobserver and intraspecific variation. Conversely, Fruciano and colleagues [18] also compared intra- and interobserver rates between two researchers using three different surface scan methodologies for a series of marsupial crania. These researchers found significant differences in landmark protocols *both* between observers and among the different scan types, and found that the differences in landmark collection protocols led to statistically different results when estimating phylogenetic signal in their dataset.

These studies demonstrate that training and a consistently applied protocol could reduce some technological and user-based error, although many of these results are contradictory. All previous studies thus far fail to address the possibility that in-person training may be impractical or impossible in some cases, and they use only three scan types while a wide variety of scanners is currently available on the market. Additionally, with the involvement of many more researchers of varying expertise levels, this study will provide more robust results regarding the magnitude of potential interobserver error.

As landmark-based studies increasingly move toward the use of surface scanners for creating virtual specimens of fossil (e.g., [19,20, 21, 22]) and extant (e.g., [23, 24, 25]) organisms that can be archived for sharing and future use, questions addressing the compatibility of data collected by different researchers with inherently different methods and equipment are paramount if truly collaborative and accurate research is to be achieved. Quantifying and understanding how intra- and interobserver error are affected by both technology and user error is especially relevant now as data sharing efforts are becoming common in the paleoanthropology and paleontology communities through open-access web databases like PRIMO (http://primo.nycep.org) and MorphoSource (www.morphosource.org), where both morphometric data and raw scans are shared freely among researchers.

Given the multiple potential sources of error in any landmark-based study, our goal here is to investigate whether landmarks can be placed at truly homologous points given the inherent differences in researcher experience, landmarking techniques, and the quality of a digital model resulting from different scanners and scanning protocols. To evaluate the gravity of some of these issues, we assess the compatibility of landmark data gathered by nine researchers with varying degrees of experience on scans of a single macaque cranium digitally rendered by four different scanners (see Table 1). We apply multivariate statistics to evaluate rates of precision and accuracy among researchers, and test the following three predictions:

**Table 1:** List of scanners and scanner types used for this project. Faces refers to the number of triangles in a surface.

Scanner name	Type (abbreviations used in later tables)	Scanner resolution	Scan surface area (mm^2^) / volume (mm^3^)
NextEngine, Inc. NextEngine 3D Scanner HD	Laser surface scanner (NE)	0.1 mm	47,075 / 208,180
Breuckmann OptoTOP-HE	Structured white light surface scanner ((B)	2 μm	46,085 / 256,581
Minolta Vivid 910	Laser surface scanner (M)	1.12 mm	49,000 / 275,592
General Electric Phoenix v|tome|x s240	Computed Tomography (CT)	< 1 μm	5,905,620 / 566,477

1. Higher scan quality (as determined by higher resolution and point density) will reduce both intra- and interobserver error.
  We here aim to test if the differences in surface rendering inherent to different scanners will influence the ability of a researcher to both precisely and accurately landmark a digital scan model. We predict that higher scan quality will enable researchers to more accurately and precisely landmark digital specimens, regardless of training or levels of experience.
2. Increased experience with 3DGM and/or osteology will decrease both intra- and interobserver error.
  We here assess whether experience positively correlates with both accuracy and precision in the ability of a researcher to apply landmarks to a 3D model. We predict that users with more osteological and morphometric experience will have lower rates of intraobserver error, and also that rates of interobserver error will be significantly less among these experienced individuals. We expect researchers with low levels of experience to have high rates of both inter- and intraobserver error. We predict a positive correlation with experience and precision/accuracy.
3. In-person training provided by a single, experienced researcher will decrease both intra- and interobserver error rates of researchers that receive it.
  We here test whether personal instruction on how to collect landmarks has any influence on rates of variance. We predict that training will cause a reduction in interobserver error among those individuals that received it, and that it will significantly reduce intraobserver error for those trained individuals as compared to those without in-person training.

Finally, we also evaluate the efficacy of sliding semilandmarks for inter- and intraobserver error reduction.

## Materials and methods

### Materials

**Fig 1:**
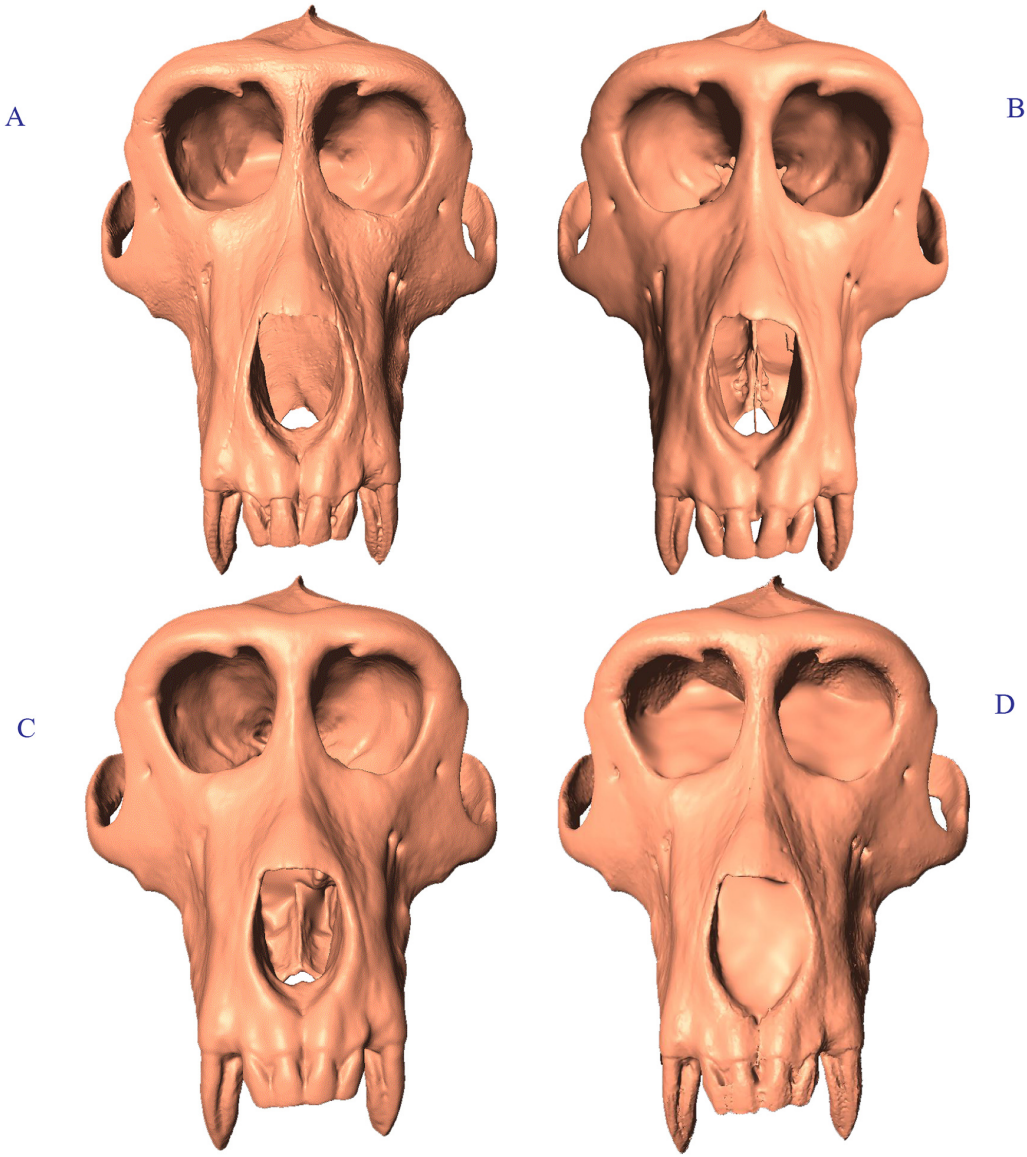
Scan comparison anterior view of *Macaca thibetana* (AMNH 129). (A) Breuckmann OptoTOP-HE; (B) GE Phoenix v|tome|x s240 CT scan; (C) Minolta Vivid 910; (D) NextEngine 3D Scanner HD.

**Fig 2:**
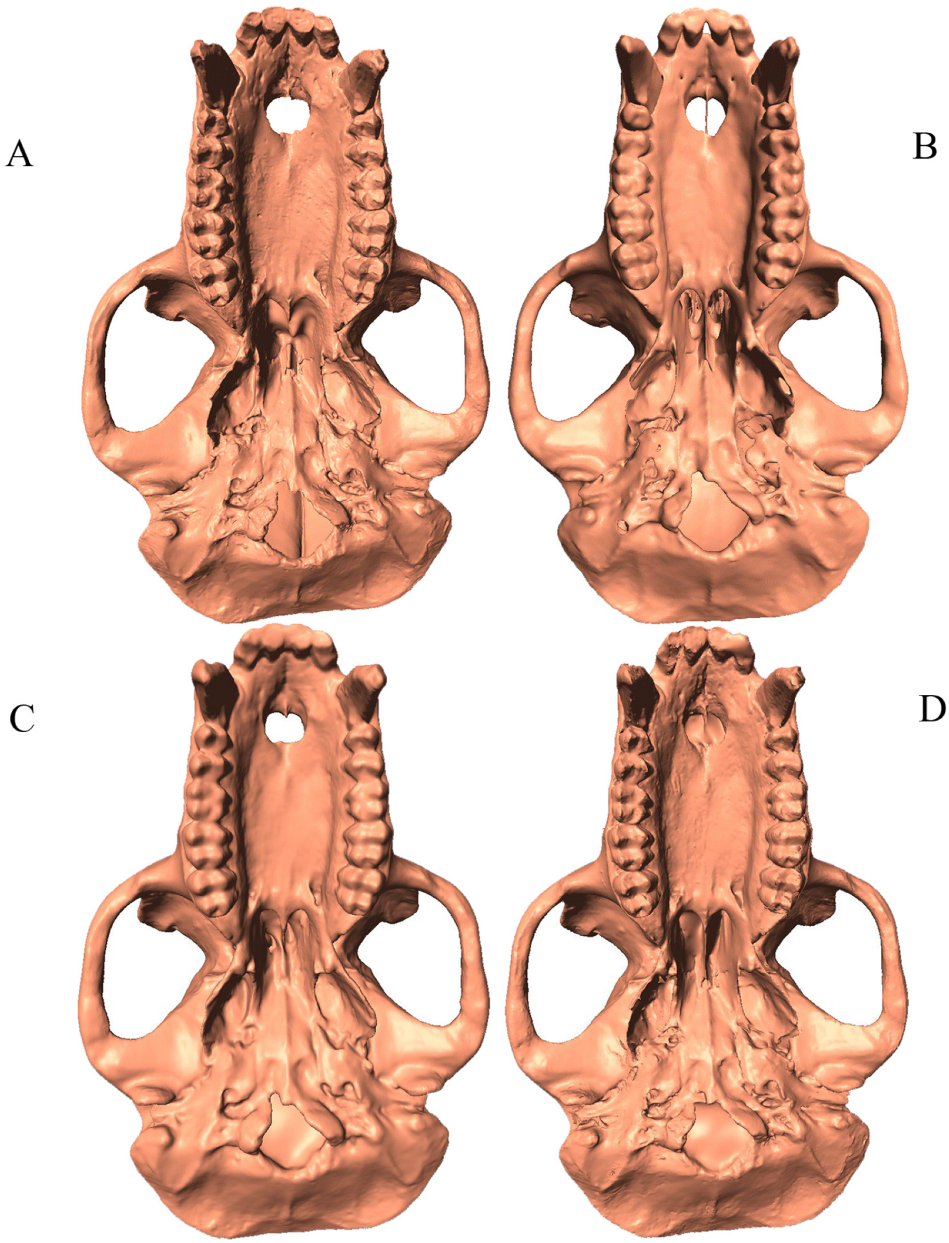
Scan comparison inferior view of *Macaca thibetana* (AMNH 129). (A). Breuckmann OptoTOP-HE; (B) GE phoenix v|tome|x s240 CT scan; (C) Minolta Vivid 910; (D) NextEngine 3D Scanner HD.

Digital models of an adult male Tibetan macaque (*Macaca thibetan(A)* cranium (American Museum of Natural History [AMNH] Mammalogy Department 129) were generated with two laser surface scanners (NextEngine Desktop 3D Scanner HD and Minolta Vivid 910), a structured white light scanner (the Breuckmann OptoTOP-HE), and a computed tomography (CT) scanner (General Electric Phoenix v|tom|x s240) (See Table 1; Figs 1 and 2). Laser surface scans were digitally processed in 2011 using Geomagic Studio 12 (now 3D Systems), white light scans were processed in OPTOCAT (the native Breuckmann editing software package), and CT scans were processed using VGStudio Max (Volume Graphics). For surface scans, post-processing was limited to the removal of extraneous material digitized by the scanner (e.g., the turntable on which the specimen was placed, any modeling clay used for support, etc.), curve-based hole filling, and refinement of minor mesh artifacts unavoidably generated during the scanning process (e.g., small spikes and poorly fitted surfaces).

### Methods

**Fig 3:**
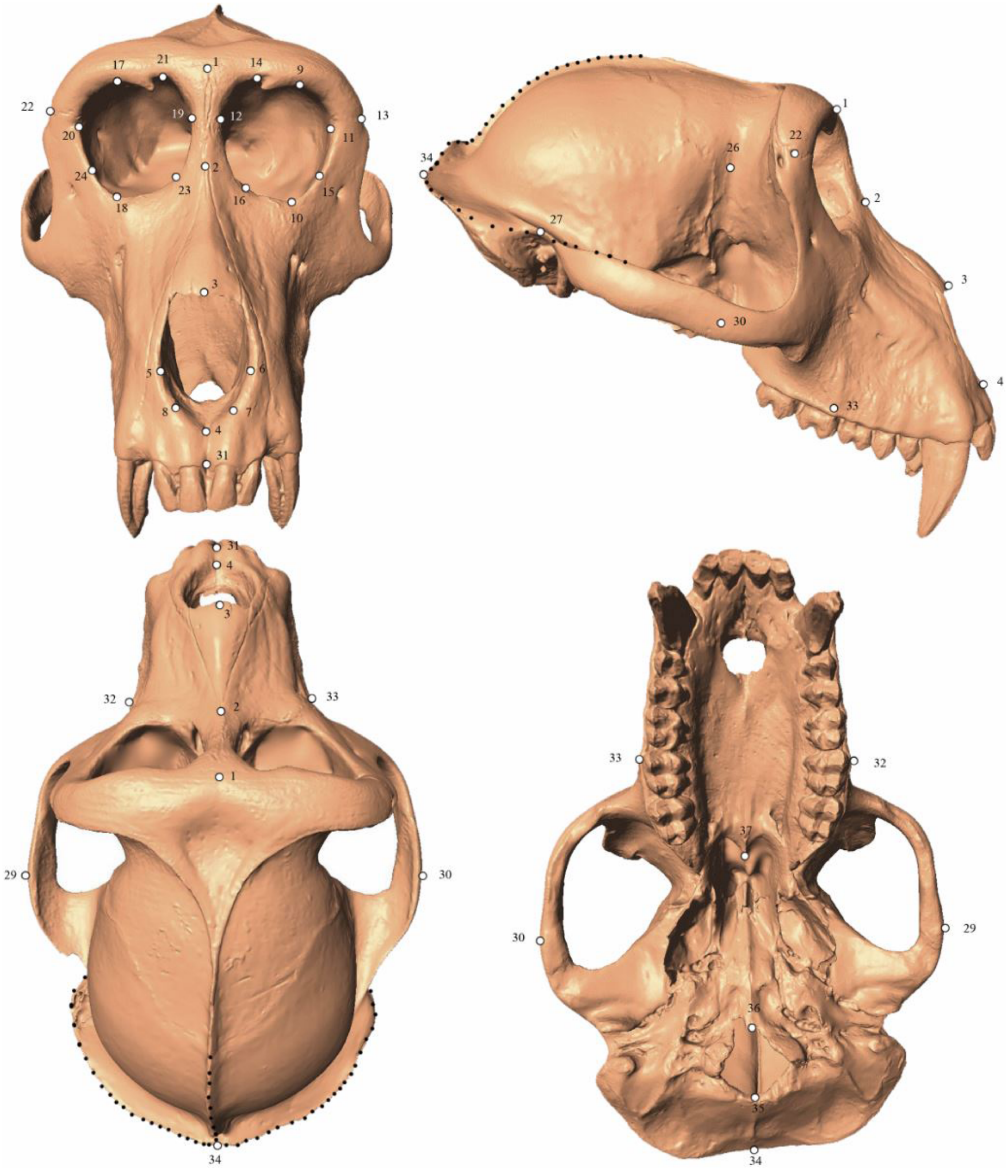
Landmarks employed in this study. Digital rendering of an adult male *Macaca thibetana* cranium (AMNH Mammalogy 129) with points depicting the 37 single landmarks (white dots) and three curves (black dotted lines) used in this study.

Scans were imported into the program Landmark Editor [2] where nine researchers (hereafter referred to as R1, R2, R3, etc.) with varying degrees of expertise as denoted by the suffixes (LX) for low experience, (MX) for medium experience, (HX) for high experience, and (T) for trainer (Table 2) placed thirty-seven Type I, II, and III landmarks and three three-dimensional semilandmark curves (Fig 3). The experience designation is based on the overall osteological knowledge and prior exposure to 3D geometric morphometrics methods. Each semilandmark curve was defined using three Type I, II or III landmarks as “anchors”; a series of 10 semilandmarks were automatically generated equidistant from one another along that curve (see Fig 3 and Table 3). The application of semilandmark curves was independent of other landmarks, even though they may share a point as an “anchor”, as Landmark Editor allows for the joining of multiple curves. This dataset was designed to reflect commonly used osteometric points and to cover often-studied areas of the cranium. All researchers who landmarked crania were given a written description of the landmark points (see Table 3), and an illustration of the points as defined by R9. For the researchers trained in person by R9, a pre-landmarked “atlas” cranium was included each project file to serve as a reference for those with less osteological experience and R9 was available to answer any questions and give clarifications. No additional assistance was given beyond these tools during the landmarking trials.

**Table 2:** List of observers who collected data, their experience, and the order in which they landmarked the scan replicates (scanner abbreviations from Table 1). Each observer is designated by both a number (e.g., R1, R2, R3) and an experience abbreviation: LX = low experience, MX = medium experience, HX = High experience, T = Trainer. Experience designations were assigned based on overall osteological knowledge and familiarity with 3D GM methods and practice.

Observer	User experience		Order
Researcher 1 R1 (LX)	AMNH volunteer; undergraduate experience in osteology; first time collecting 3DGM data; received in-person instruction from R9 (T) in how to collect the data		M, CT, NE, B
Researcher 2 R2 (MX)	AMNH volunteer; undergraduate experience in osteology; 1 year of experience collecting 3DGM data; received in-person instruction from R9 (T) in how to collect the data		CT, B, NE, M
Researcher 3 R3 (LX)	AMNH volunteer; undergraduate experience in osteology; first time collecting 3DGM data; received in-person instruction from R9 (T) in how to collect the data		B, NE, CT, M
Researcher 4 R4 (MX)	AMNH volunteer; undergraduate experience in osteology; 1 year of experience collecting 3DGM data; received in-person instruction from R9 (T) in how to collect the data		CT, M, NE, B
Researcher 5 R5 (HX)	Ph.D. in physical anthropology with a morphology emphasis; regular user of 3DGM data; received the list of landmark definitions but no in-person training		B, M, CT, NE
Researcher 6 R6 (HX)	Ph.D. in physical anthropology with a morphology emphasis; regular user of 3DGM data; received the list of landmark definitions but no in-person training		B, CT, M, NE
Researcher 7 R7 (MX)	AMNH volunteer; undergraduate experience in osteology; 1 year of experience collecting 3DGM data; received in-person instruction from R9 (T) in how to collect the data		M, B, CT, NE
Researcher 8 R8 (HX)	Graduate student in physical anthropology with morphology emphasis; significant experience in osteology; significant experience collecting 3DGM data; received the list of landmark definitions and in-person clarification of questions from R9 (T)		M, CT, NE, B
Researcher 9 (HX, T)	Ph.D. in physical anthropology with a morphology emphasis; regular user of 3DGM data, Trainer.		M, NE, B, CT
Low experience (LX)	Medium experience (MX)	High experience (HX)	Trainer
Researcher 1 Researcher 3	Researcher 2 Researcher 4 Researcher 7	Researcher 5 Researcher 6 Researcher 8	Researcher 9

**Table 3:** List of landmarks used in this study. Bilateral landmarks denoted by (L) and (R) for their respective anatomical sides. Quotation marks indicate identical description to point listed directly above. SLC = Semilandmark curve. For inclusion in sets, F = Full landmark set, R = Reduced landmark set, and S = Semilandmark only set. This landmark definition set and an illustrated atlas were provided to each researcher before their respective landmarking trials.

#	Osteometric Point Name	Description	Side	Landmark type	Included in Landmark Set:
1	Glabella	Most anterior point in the mid-sagittal plane between the supraciliary arches	Midline	III	F, R
2	Nasion	Point where nasals and frontal meet in midline	Midline	I	F, R
3	Rhinion	Most inferior point in midline where nasals meet		I	F, R
4	Nasiospinale	Most inferior point in midline on nasal aperture		I	F, R
5	Alare (L)	Most lateral point on nasal aperture in transverse plane	Left	III	F, R
6	Alare (R)	Most lateral point on nasal aperture in transverse plane	Right	III	F, R
7		Point of maximum curvature on inferiormost corner of nasal aperture	Left	III	F, R
8		Point of maximum curvature on inferiormost corner of nasal aperture	Right	III	F, R
9		Superior most point in lateral half of supraorbital margin	Left	III	F, R
10	Orbitale (L)	Most inferior point on infraorbital margin	Left	III	F, R
11	Ectoconchion (L)	Lateral most point on orbit in transverse plane	Left	III	F, R
12		Medial most point on orbit in transverse plane	Left	III	F, R
13	Frontomalare temporale (L)	Point where zygomatico-frontal suture crosses lateral edge of zygoma.	Left	I	F, R
14		Center of supraorbital foramen/notch	Left	II	F, R
15		Point of maximum curvature on inferolateral infraorbital margin	Left	III	F, R
16		Point of maximum curvature on inferomedial infraorbital margin	Left	III	F, R
17		Superior most point in lateral half of supraorbital margin	Right	III	F, R
18	Orbitale (R)	Most inferior point on infraorbital margin	Right	III	F, R
19		Medial most point on orbit in transverse plane	Right	III	F, R
20	Ectoconchion (R)	Lateral most point on orbit in transverse plane	Right	III	F, R
21		Center of supraorbital foramen/notch	Right	II	F, R
22	Frontomalare temporale (R	Point where zygomatico-frontal suture crosses lateral edge of zygoma	Right	I	F, R
23		Point of maximum curvature on inferomedial infraorbital margin	Right	III	F, R
24		Point of maximum curvature on inferolateral infraorbital margin	Right	III	F, R
25		Point of maximum postorbital constriction	Left	III	F
26		Point of maximum postorbital constriction	Right	III	F
27	Porion (L)	Most superolateral point of external auditory meatus	Left	III	F, R
28	Porion (R)	Most superolateral point of external auditory meatus	Right	III	F, R
29	Zygion (L)	Most lateral Point of zygomatic arch	Left	III	F
30	Zygion (R)	Most lateral Point of zygomatic arch	Right	III	F
31	Prosthion	Most anterior point of alveolar process of maxilla in midline	Midline	I	F, R
32		Widest breadth of alveolar process of maxilla	Left	III	F
33		Widest breadth of alveolar process of maxilla	Right	III	F
34	Opisthocranion	Most posterior point of cranium in midline	Midline	II	F, R
35	Opisthion	Most posterior point of foramen magnum in midline	Midline	III	F, R
36	Basion	Most anterior point of foramen magnum in midline	Midline	III	F, R
37		Most posterior point of horizontal plate of palatine bone in midline	Midline	II	F, R
38–47	Curve 1	Asterion (L) to Opisthocranion	SLC	S	F, S
48–57	Curve 2	Opisthocranion to Asterion (R)	SLC	S	F, S
58–67	Curve 3	Opisthocranion to Bregma	SLC	S	F, S

Three landmark configurations were analysed to test the relative stability and usefulness of various landmark types:

1. a “Full” landmark set consisting of all points initially described in the landmark protocol, including Type I, II, and III landmarks, and additionally a series of semilandmark curves.
2. a “Reduced” landmark set including most Type I, II and III landmarks, but with semilandmarks and the most variable Type II and III landmarks removed (Landmarks 25, 26, 29, 30, 32 and 33). This landmark set was evaluated to test the variance on only relatively ‘stable’ and easily found landmarks, thereby potentially limiting the influence of difficult to find (or easily damage(D) points on dry crania.
3. a “Semilandmark only” set consisting of only those points joined together by the curve function of Landmark Editor (points 38 through 67). These semilandmarks were applied independently from other landmarks during the initial “Full” landmark set application.

The Reduced landmark set and Semilandmark only set were created post hoc by removing points from the Full landmark set according to the specifics of each protocol as listed above, which were then independently tested to verify the influence of different point configurations. All statistical tests were performed on each of the three landmark sets in independent iterations. Additionally, the amount of variance was calculated for each individual landmark point to assess which discrete landmarks (or landmark types) are most prone to user error.

Each researcher placed the full landmark set on 10 replicates of the macaque cranium from each scanner (i.e., 10 replicates of the Breuckmann OptoTOP-HE scan, 10 replicates of the NextEngine scan, etc.) to assess variation in user accuracy and precision. Each user placed their landmarks on the different scans types in unique orders so as not to bias the results due to practice (see Table 2). The Reduced and Semilandmark only sets were subsequently analyzed by removing points prior to all relevant geometric morphometric analyses (See Table 3). Semilandmark sliding is a technique used with semilandmarks to “slide” them into their most homologous positions by either minimizing the bending energy or Procrustes distance among specimens [9, 26]. The purpose of these analyses was to assess sources of error, and all data were collected on the same cranium; therefore, sliding semilandmark protocols were not employed here as there are no issues with homology between specimens.

Landmark coordinates were exported to *morphologika* v2.5 [27] which was used to perform a generalized Procrustes analysis (GPA). This analysis translates, scales, and rigidly rotates specimen configurations around a common centroid, using a least-squares algorithm to optimally minimize the distance each shape lies from the origin [28,29,30]. A separate GPA was performed for each observer to assess inter-scan error and intraobserver error. A GPA of the entire pooled dataset was used to assess interobserver error.

In addition to landmarking replicates of the same cranium, Researchers 6 (HX) and 8 (HX) placed the full landmark configuration on a total of 10 female macaque crania from 7 different species to compare the magnitude of interobserver error to normal species and inter-species shape differences (see Table 4). Steps of this second data collection were identical to those previously listed for the adult female *M*. *thibetana* cranium (AMNH Mammalogy 129). In this instance, all analyses were performed both with and without sliding the semilandmarks as there were different crania as part of the dataset. For this analysis including specimens of multiple taxa, semilandmarks were slid into their most homologous positions by minimizing the Procrustes distances among the specimens. All analyses were completed in the *geomorph* package for R [31].

**Table 4:** Sample of *Macaca* used for testing the magnitude of interobserver error.

Taxon	N	Specimen numbers
*Macaca mulatta*	1	NMNH (National Museum of Natural History) 173813
*Macaca nemestrina*	2	AMNH 11090, 106037
*Macaca nigra*	1	AMNH 196414
*Macaca ochreata*	1	AMNH 153599
*Macaca sylvanus*	2	NMNH 476780, 476785
*Macaca thibetana*	1	AMNH 83994
*Macaca tonkeana*	2	AMNH 152907, 153401

#### Effects of landmark position on error

The variance for each individual landmark was assessed by computing the average Procrustes distance between the mean landmark position and each individual replicate for each researcher. In this instance, the data collected by each researcher were subject to a separate GPA. The variance for each landmark was also calculated for the entire dataset. In this case, all data from all users were subjected to a single GPA and the same process was followed for computing the mean error for each landmark.

#### Effects of scan type on error

The amount of intraobserver error per scan type was calculated for each individual for each landmark configuration. Intraobserver error was calculated as the Procrustes distance (defined as the square root of the sum of squares distances between corresponding landmarks of shapes after superimposition [9]) between each replicate and the mean for all replicates for each scan from a single researcher. Significant differences in error among scan types were assessed using an ANOVA with Tukey’s pairwise post hoc comparisons to determine whether intraobserver error was significantly lower for any particular scanner. Box plots were generated in PAST v 3.0 [32] to illustrate differences in variance among scan types for each researcher; solid lines indicate median variance, the boxes indicate the 25–75% quartile, and the whiskers extend to the farthest data point that is less than 1.5x the height of the box. Finally, all Procrustes distances from the mean from all nine researchers for each scan type were pooled. A boxplot illustrating the distribution of distances for each scan type was produced in PAST [32]. An ANOVA with Tukey’s post hoc comparison was performed to determine if there was an overall mean difference in rates of intraobserver error among the scan types. A two-way ANOVA with Tukey’s post hoc pairwise comparisons was performed to determine whether there were significant differences between scan types when differences among researchers were also part of the model.

The amount of interobserver error for each scan type was recorded as the series of pairwise Procrustes distances between all different users for each scanner. Boxplots were created using PAST [32] to illustrate the range of pairwise Procrustes distances. Significant differences among the ranges of pairwise Procrustes distances were tested using an ANOVA with Tukey’s post hoc pairwise comparisons.

#### Effects of experience on error

To compare the degree of intraobserver error among researchers, we examined the total intraobserver error for each individual using the range of Procrustes distances from the mean using all forty replicates. Box plots of these data were generated in PAST [32] to illustrate differences in intraobserver error among users as described previously. An ANOVA with Tukey’s post hoc pairwise comparisons was performed to determine if there were significant differences among users in the degree of intraobserver error.

In order to explore whether experience influenced patterns of intraobserver error, principal components analyses (PC(A) were generated with MorphoJ [33]. Percent variance on the first three axes was also recorded. If the percent variance accounted for by each axis is low, variation in landmark placement is occurring isotropically as variance is occurring in many different directions. If percent variance is high on the first axis, it indicates that error is occurring anisotropically for certain landmarks.

#### Effects of training on error

A PCA of the Procrustes aligned coordinates for all trials for all users was performed and the first two principal components were visualized. If in-person training had a positive effect on landmark consistency, those individuals who received training should appear in a common area of the morphospace. In addition, a UPGMA dendrogram constructed using average Procrustes distances among researchers was also created using PAST [32] to see if users receiving in-person training formed a single cluster.

#### Interobserver error vs. shape variability in multiple species

Interobserver error was calculated as the Procrustes distance between each replicate and the mean of the entire dataset. To assess whether rates of interobserver error (with and without training) were larger than a real biological signal, the pooled interobserver error rates for all researchers and trials on the single *M*. *thibetana* cranium were plotted in three boxplots with the pooled error rates for the seven different macaque species landmarked by R6 (HX) and R8 (HX).

## Results

### Effects of landmark type on error

The results for intra- and interobserver error at each landmark are presented in Table 5. In terms of intraobserver error, there was no discernable pattern for which landmarks were *always* the most or least error prone. However, Landmarks 25, 26, 29 and 30 commonly had relatively high levels of intraobserver error. Landmark 3 had one of the lowest intraobserver errors in seven out of nine researchers, and landmarks 14, 21 and 35 also commonly had relatively low levels of intraobserver error. There were six landmarks that had much higher interobserver errors when compared to all of the other landmarks. Those landmarks were 25, 26, 29, 30, 32 and 33 and were removed from the Reduced landmark configuration in all subsequent analyses. These are all Type III landmarks and as such were expected to be the most error prone.

**Table 5:** Average Procrustes distance from the centroid to each replicate for every Type I, II or III landmark in the analysis. Data for individual Procrustes alignment indicate that only the 40 replicates for each individual were used in the calculation; full Procrustes alignment includes all replicates for all individuals in a single Procrustes alignment. Bolded values indicate the six largest average Procrustes distances for the alignment using all users; these were the landmarks removed in the Reduced Landmark dataset.

#	Individual Procrustes alignments									Procrustes alignment—All users
	R1 (LX)	R2 (MX)	R3 (LX)	R4 (MX)	R5 (HX)	R6 (HX)	R7 (MX)	R8 (HX)	R9 (T)	
1	0.006	0.007	0.006	0.003	0.004	0.005	0.019	0.005	0.009	0.017
2	0.004	0.007	0.005	0.005	0.003	0.009	0.005	0.008	0.009	0.014
3	0.002	0.004	0.003	0.002	0.001	0.002	0.006	0.002	0.002	0.006
4	0.004	0.005	0.005	0.003	0.003	0.005	0.009	0.003	0.003	0.009
5	0.003	0.006	0.004	0.003	0.003	0.003	0.006	0.004	0.003	0.009
6	0.004	0.006	0.004	0.004	0.003	0.002	0.006	0.004	0.004	0.008
7	0.003	0.005	0.004	0.002	0.004	0.003	0.008	0.004	0.003	0.008
8	0.004	0.005	0.006	0.003	0.004	0.002	0.008	0.003	0.004	0.009
9	0.009	0.004	0.004	0.002	0.003	0.002	0.004	0.003	0.004	0.008
10	0.011	0.006	0.004	0.003	0.002	0.002	0.005	0.004	0.004	0.008
11	0.003	0.004	0.004	0.004	0.003	0.003	0.005	0.004	0.010	0.008
12	0.003	0.005	0.005	0.003	0.003	0.003	0.005	0.005	0.006	0.007
13	0.008	0.006	0.005	0.004	0.003	0.002	0.006	0.003	0.004	0.011
14	0.006	0.003	0.003	0.002	0.002	0.002	0.005	0.005	0.004	0.006
15	0.011	0.003	0.004	0.003	0.003	0.002	0.006	0.003	0.006	0.007
16	0.007	0.005	0.004	0.003	0.004	0.002	0.006	0.007	0.003	0.007
17	0.004	0.005	0.006	0.002	0.002	0.002	0.004	0.004	0.004	0.007
18	0.009	0.008	0.005	0.002	0.003	0.002	0.005	0.003	0.004	0.009
19	0.003	0.004	0.003	0.003	0.002	0.003	0.005	0.005	0.004	0.006
20	0.004	0.005	0.005	0.003	0.004	0.002	0.005	0.004	0.012	0.009
21	0.003	0.003	0.004	0.002	0.002	0.002	0.005	0.007	0.004	0.006
22	0.006	0.007	0.005	0.004	0.003	0.002	0.005	0.003	0.004	0.010
23	0.009	0.007	0.009	0.002	0.003	0.003	0.009	0.011	0.005	0.009
24	0.006	0.005	0.005	0.002	0.003	0.003	0.005	0.004	0.005	0.007
25	0.008	0.007	0.008	0.007	0.003	0.005	0.011	0.003	0.011	**0.025**
26	0.007	0.006	0.007	0.007	0.003	0.005	0.012	0.005	0.011	**0.024**
27	0.006	0.005	0.006	0.003	0.004	0.002	0.007	0.004	0.006	0.008
28	0.006	0.004	0.006	0.004	0.003	0.002	0.007	0.003	0.005	0.008
29	0.008	0.008	0.013	0.006	0.003	0.009	0.012	0.008	0.007	**0.025**
30	0.006	0.007	0.012	0.006	0.003	0.006	0.011	0.006	0.007	**0.025**
31	0.004	0.004	0.004	0.003	0.003	0.003	0.008	0.003	0.005	0.009
32	0.010	0.013	0.007	0.003	0.002	0.004	0.030	0.003	0.008	**0.032**
33	0.007	0.012	0.006	0.004	0.002	0.004	0.031	0.003	0.009	**0.032**
34	0.007	0.007	0.008	0.003	0.002	0.002	0.010	0.005	0.004	0.014
35	0.004	0.003	0.005	0.002	0.002	0.002	0.005	0.003	0.002	0.008
36	0.002	0.004	0.005	0.002	0.002	0.002	0.005	0.003	0.003	0.007
37	0.003	0.004	0.004	0.003	0.003	0.004	0.005	0.003	0.004	0.009

### The effects of scan type on error

**Fig 4:**
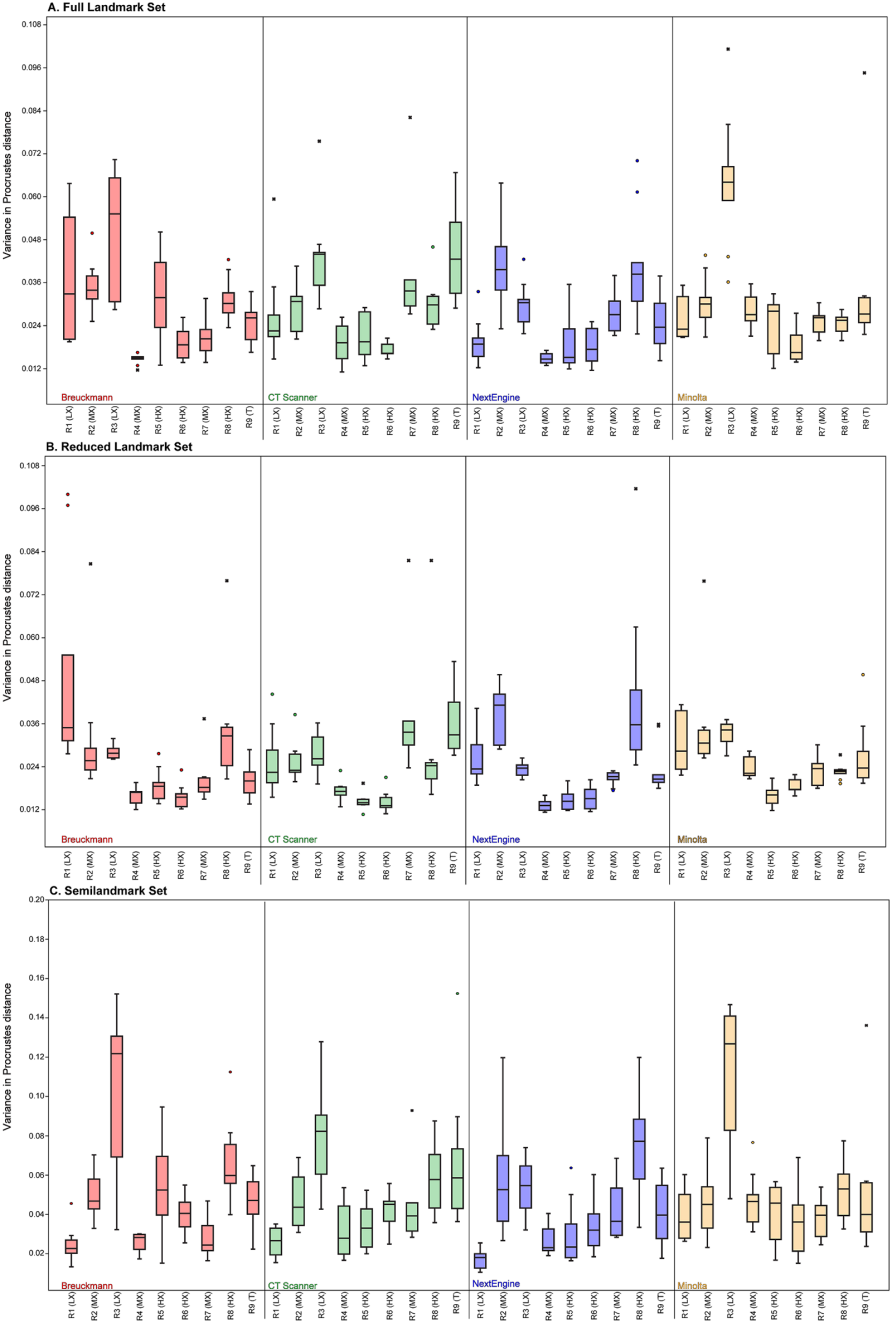
Box plot illustrating the amount of intraobserver error for each user with each scanner using each landmark set. (A) Full landmark set; (B) Reduced landmark set; (C) Semilandmark set. See Table 6 for numerical data.

**Fig 5:**
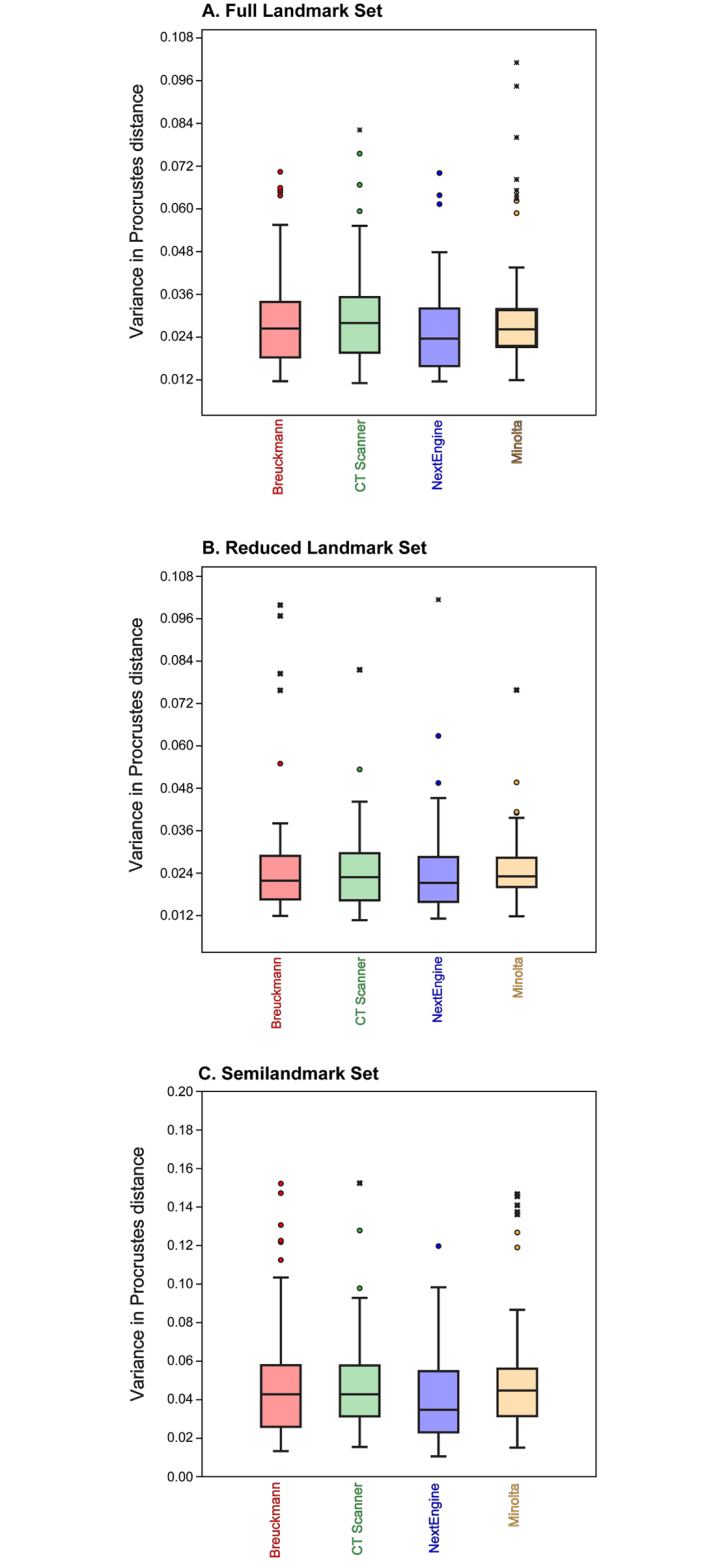
Box plot illustrating the amount of intraobserver error for each scanner type for each landmark set. (A) Full landmark set; (B) Reduced landmark set; (C) Semilandmark set.

Table 6 tabulates the average Procrustes distances from the mean shape among replicates for each user and each scan type for all three landmark configurations. These results can also be visualized as box plots in Fig 4. The results from one way ANOVAs indicate that there were some significant differences in variance among the scan types for a single researcher; however, post hoc pairwise comparisons revealed no consistent pattern explaining which pairs of scan types were significantly different from one another. Some users exhibited a trend toward similar levels of variance for scans which were landmarked in sequential order (R1 (LX), R3 (LX), and R4 (MX)), while others (R2 (MX), R3 (LX), R6 (HX), R7 (MX), and R8 (HX)) exhibited no discernible pattern in their landmarking variability. When all trials from all researchers were pooled, results of ANOVAs showed that there were no significant differences present among scanning types (p = 0.12 for the Full configuration, p = 0.88 for the Reduced configuration and p = 0.13 for the Semilandmark only configuration; Fig 5 and Tables 7–9). Thus, average intraobserver error was statistically uniform across scan types and for all three landmark configurations when users are considered as one group.

**Table 6:** Average variance for intraobserver trials for different scan types for the entire landmark protocol.

Researcher	Landmark Set	NextEngine	Breuckmann	Minolta	CT	Total average variance by Landmark set
R1 (LX)	Full	0.019	0.031	0.026	0.026	0.034
	Reduced	0.026	0.034	0.030	0.025	0.042
	Semilandmark	0.017	0.024	0.038	0.026	0.038
R2 (MX)	Full	0.040	0.035	0.030	0.029	0.040
	Reduced	0.039	0.032	0.035	0.025	0.038
	Semilandmark	0.057	0.050	0.044	0.047	0.055
R3 (LX)	Full	0.015	0.051	0.064	0.043	0.052
	Reduced	0.013	0.028	0.033	0.027	0.034
	Semilandmark	0.053	0.101	0.110	0.077	0.091
R4 (MX)	Full	0.019	0.015	0.028	0.019	0.025
	Reduced	0.015	0.016	0.023	0.017	0.021
	Semilandmark	0.026	0.026	0.047	0.030	0.041
R5 (HX)	Full	0.019	0.032	0.023	0.021	0.037
	Reduced	0.015	0.019	0.016	0.014	0.020
	Semilandmark	0.030	0.053	0.039	0.033	0.061
R6 (HX)	Full	0.018	0.019	0.019	0.017	0.022
	Reduced	0.015	0.016	0.019	0.014	0.022
	Semilandmark	0.034	0.040	0.036	0.041	0.041
R7 (MX)	Full	0.028	0.021	0.025	0.042	0.052
	Reduced	0.021	0.020	0.023	0.041	0.040
	Semilandmark	0.043	0.027	0.037	0.047	0.061
R8 (HX)	Full	0.040	0.031	0.025	0.030	0.034
	Reduced	0.043	0.034	0.023	0.028	0.035
	Semilandmark	0.075	0.066	0.051	0.058	0.066
R9 (T)	Full	0.026	0.024	0.033	0.043	0.038
	Reduced	0.023	0.020	0.027	0.036	0.037
	Semilandmark	0.038	0.046	0.050	0.068	0.057
Total average variance by scanner for all users and landmark sets	Full	0.026	0.029	0.031	0.030	0.0288
	Reduced	0.024	0.026	0.025	0.025	0.0252
	Semilandmark	0.041	0.048	0.05	0.48	0.0468

**Table 7:** Results of a one-way ANOVA for scanner for the Full data set.

	Sum of Squares	df	Mean Square	F	p-value
Between Groups	.001	3	.000	1.957	.120
Within Groups	.072	356	.000		
Total	.073	359			

**Table 8:** One-way ANOVA for scanner of the Reduced landmark dataset.

	Sum of Squares	df	Mean Square	F	p-value
Between Groups	.000	3	.000	.225	.879
Within Groups	.061	356	.000		
Total	.061	359			

**Table 9:** One-way ANOVA for scanner of the Semilandmark data set.

	Sum of Squares	df	Mean Square	F	p-value
Between Groups	.004	3	.001	1.843	.139
Within Groups	.255	356	.001		
Total	.259	359			

When both user and scanner are taken into account, two-way ANOVAs show that there is a significant difference in levels of intraobserver error between the NextEngine and both the CT and Minolta scanners for the Full and Semilandmark data sets (Tables 10–18). However, the effect size (as measured by the mean difference in intraobserver error between scanners) is smaller than the average intraobserver error for any user (Table 6). There is no significant difference among scanners for the Reduced landmark dataset.

**Table 10:** Results of a two-way ANOVA for user and scanner for the Full landmark set.

Source	Type III Sum of Squares	df	Mean Square	F	p-value
Corrected Model	.041	35	.001	11.688	p<0.001
Intercept	.299	1	.299	3005.062	p<0.001
Scanner	.001	3	.000	3.965	.008
User	.024	8	.003	30.201	p<0.001
Scanner User	.015	24	.001	6.483	p<0.001
Error	.032	324	.000		
Total	.372	360			
Corrected Total	.073	359			

**Table 11:** Tukey’s post hoc pairwise comparisons for scanners for the Full landmark set.

(I) scanner	(J) scanner	Mean Difference (I-J)	p-value	95% Confidence Interval	
				Lower Bound	Upper Bound
BR	CT	-.0009	.939	-.0047	.0030
	M	-.0013	.817	-.0051	.0025
	NE	.0033	.116	-.0005	.0072
CT	BR	.0009	.939	-.0030	.0047
	M	-.0004	.991	-.0043	.0034
	NE	.0042	.027	.0003	.0080
M	BR	.0013	.817	-.0025	.0051
	CT	.0004	.991	-.0034	.0043
	NE	.0046	.011	.0008	.0085
NE	BR	-.0033	.116	-.0072	.0005
	CT	-.0042	.027	-.0080	-.0003
	M	-.0046	.011	-.0085	-.0008

**Table 12:** Tukey’s post hoc pairwise comparisons for users for the Full landmark set.

(I) user	(J) user	Mean Difference (I-J)	p-value	95% Confidence Interval	
				Lower Bound	Upper Bound
R1 (LX)	R8 (HX)	.0049	.408	-.0021	.0119
	R2 (MX)	-.0021	.991	-.0090	.0049
	R3 (LX)	-.0154	p<0.001	-.0224	-.0084
	R7 (MX)	.0026	.959	-.0043	.0096
	R5 (HX)	.0077	.017	.0008	.0147
	R9 (T)	.0005	1.000	-.0065	.0075
	R4 (MX)	.0124	p<0.001	.0054	.0193
	R6 (HX)	.0134	p<0.001	.0065	.0204
R8 (HX)	R1 (LX)	-.0049	.408	-.0119	.0021
	R2 (MX)	-.0070	.050	-.0139	.0000
	R3 (LX)	-.0203	p<0.001	-.0273	-.0133
	R7 (MX)	-.0023	.984	-.0092	.0047
	R5 (HX)	.0028	.940	-.0041	.0098
	R9 (T)	-.0044	.558	-.0114	.0025
	R4 (MX)	.0074	.026	.0005	.0144
	R6 (HX)	.0085	.005	.0015	.0155
R2 (MX)	R1 (LX)	.0021	.991	-.0049	.0090
	R8 (HX)	.0070	.050	.0000	.0139
	R3 (LX)	-.0133	p<0.001	-.0203	-.0064
	R7 (MX)	.0047	.467	-.0023	.0117
	R5 (HX)	.0098	.001	.0028	.0168
	R9 (T)	.0026	.967	-.0044	.0095
	R4 (MX)	.0144	p<0.001	.0075	.0214
	R6 (HX)	.0155	p<0.001	.0085	.0224
R3 (LX)	R1 (LX)	.0154	p<0.001	.0084	.0224
	R8 (HX)	.0203	p<0.001	.0133	.0273
	R2 (MX)	.0133	p<0.001	.0064	.0203
	R7 (MX)	.0180	p<0.001	.0111	.0250
	R5 (HX)	.0231	p<0.001	.0162	.0301
	R9 (T)	.0159	p<0.001	.0089	.0228
	R4 (MX)	.0277	p<0.001	.0208	.0347
	R6 (HX)	.0288	p<0.001	.0218	.0358
R7 (MX)	R1 (LX)	-.0026	.959	-.0096	.0043
	R8 (HX)	.0023	.984	-.0047	.0092
	R2 (MX)	-.0047	.467	-.0117	.0023
	R3 (LX)	-.0180	p<0.001	-.0250	-.0111
	R5 (HX)	.0051	.357	-.0019	.0120
	R9 (T)	-.0022	.989	-.0091	.0048
	R4 (MX)	.0097	.001	.0027	.0167
	R6 (HX)	.0108	p<0.001	.0038	.0177
R5 (HX)	R1 (LX)	-.0077	.017	-.0147	-.0008
	R8 (HX)	-.0028	.940	-.0098	.0041
	R2 (MX)	-.0098	.001	-.0168	-.0028
	R3 (LX)	-.0231	p<0.001	-.0301	-.0162
	R7 (MX)	-.0051	.357	-.0120	.0019
	R9 (T)	-.0072	.034	-.0142	-.0003
	R4 (MX)	.0046	.493	-.0023	.0116
	R6 (HX)	.0057	.213	-.0013	.0126
R9 (T)	R1 (LX)	-.0005	1.000	-.0075	.0065
	R8 (HX)	.0044	.558	-.0025	.0114
	R2 (MX)	-.0026	.967	-.0095	.0044
	R3 (LX)	-.0159	p<0.001	-.0228	-.0089
	R7 (MX)	.0022	.989	-.0048	.0091
	R5 (HX)	.0072	.034	.0003	.0142
	R4 (MX)	.0119	p<0.001	.0049	.0188
	R6 (HX)	.0129	p<0.001	.0060	.0199
R4 (MX)	R1 (LX)	-.0124	p<0.001	-.0193	-.0054
	R8 (HX)	-.0074	.026	-.0144	-.0005
	R2 (MX)	-.0144	p<0.001	-.0214	-.0075
	R3 (LX)	-.0277	p<0.001	-.0347	-.0208
	R7 (MX)	-.0097	.001	-.0167	-.0027
	R5 (HX)	-.0046	.493	-.0116	.0023
	R9 (T)	-.0119	p<0.001	-.0188	-.0049
	R6 (HX)	.0011	1.000	-.0059	.0080
R6 (HX)	R1 (LX)	-.0134	p<0.001	-.0204	-.0065
	R8 (HX)	-.0085	.005	-.0155	-.0015
	R2 (MX)	-.0155	p<0.001	-.0224	-.0085
	R3 (LX)	-.0288	p<0.001	-.0358	-.0218
	R7 (MX)	-.0108	p<0.001	-.0177	-.0038
	R5 (HX)	-.0057	.213	-.0126	.0013
	R9 (T)	-.0129	p<0.001	-.0199	-.0060
	R4 (MX)	-.0011	1.000	-.0080	.0059

**Table 13:** Results of a two-way ANOVA for the Reduced landmark dataset.

Source	Type III Sum of Squares	df	Mean Square	F	p-value
Corrected Model	.028	35	.001	7.938	p < 0.001
Intercept	.228	1	.228	2252.955	p < 0.001
scanner	.000	3	.000	.379	.768
user	.016	8	.002	19.504	p < 0.001
scanner user	.012	24	.001	5.028	p < 0.001
Error	.033	324	.000		
Total	.289	360			
Corrected Total	.061	359			

**Table 14:** Tukey’s post hoc pairwise comparisons for scanners for the Reduced landmark set.

(I) scanner	(J) scanner	Mean Difference (I-J)	p-value	95% Confidence Interval	
				Lower Bound	Upper Bound
BR	CT	.0004	.995	-.0035	.0042
	M	.0004	.994	-.0035	.0043
	NE	.0015	.747	-.0024	.0054
CT	BR	-.0004	.995	-.0042	.0035
	M	.0000	1.000	-.0038	.0039
	NE	.0011	.870	-.0027	.0050
M	BR	-.0004	.994	-.0043	.0035
	CT	.0000	1.000	-.0039	.0038
	NE	.0011	.877	-.0027	.0050
NE	BR	-.0015	.747	-.0054	.0024
	CT	-.0011	.870	-.0050	.0027
	M	-.0011	.877	-.0050	.0027

**Table 15:** Tukey’s post hoc pairwise comparisons for users for the Reduced landmark set.

(I) user	(J) user	Mean Difference (I-J)	p-value	95% Confidence Interval	
				Lower Bound	Upper Bound
R1 (LX)	R8 (HX)	-.0004	1.000	-.0075	.0066
	R2 (MX)	-.0006	1.000	-.0076	.0064
	R3 (LX)	.0040	.709	-.0031	.0110
	R7 (MX)	.0056	.242	-.0014	.0126
	R5 (HX)	.0160	p < 0.001	.0090	.0231
	R9 (T)	.0055	.274	-.0016	.0125
	R4 (MX)	.0145	p < 0.001	.0075	.0216
	R6 (HX)	.0160	p < 0.001	.0090	.0230
R8 (HX)	R1 (LX)	.0004	1.000	-.0066	.0075
	R2 (MX)	-.0002	1.000	-.0072	.0069
	R3 (LX)	.0044	.573	-.0026	.0114
	R7 (MX)	.0060	.157	-.0010	.0131
	R5 (HX)	.0165	p < 0.001	.0095	.0235
	R9 (T)	.0059	.180	-.0011	.0129
	R4 (MX)	.0150	p < 0.001	.0080	.0220
	R6 (HX)	.0164	p < 0.001	.0094	.0234
R2 (MX)	R1 (LX)	.0006	1.000	-.0064	.0076
	R8 (HX)	.0002	1.000	-.0069	.0072
	R3 (LX)	.0046	.527	-.0025	.0116
	R7 (MX)	.0062	.134	-.0008	.0132
	R5 (HX)	.0166	p < 0.001	.0096	.0237
	R9 (T)	.0061	.155	-.0010	.0131
	R4 (MX)	.0151	p < 0.001	.0081	.0222
	R6 (HX)	.0166	p < 0.001	.0095	.0236
R3 (LX)	R1 (LX)	-.0040	.709	-.0110	.0031
	R8 (HX)	-.0044	.573	-.0114	.0026
	R2 (MX)	-.0046	.527	-.0116	.0025
	R7 (MX)	.0016	.998	-.0054	.0087
	R5 (HX)	.0121	p < 0.001	.0051	.0191
	R9 (T)	.0015	.999	-.0055	.0085
	R4 (MX)	.0106	p < 0.001	.0036	.0176
	R6 (HX)	.0120	p < 0.001	.0050	.0190
R7 (MX)	R1 (LX)	-.0056	.242	-.0126	.0014
	R8 (HX)	-.0060	.157	-.0131	.0010
	R2 (MX)	-.0062	.134	-.0132	.0008
	R3 (LX)	-.0016	.998	-.0087	.0054
	R5 (HX)	.0105	p < 0.001	.0034	.0175
	R9 (T)	-.0001	1.000	-.0072	.0069
	R4 (MX)	.0090	.003	.0019	.0160
	R6 (HX)	.0104	p < 0.001	.0034	.0174
R5 (HX)	R1 (LX)	-.0160	p < 0.001	-.0231	-.0090
	R8 (HX)	-.0165	p < 0.001	-.0235	-.0095
	R2 (MX)	-.0166	p < 0.001	-.0237	-.0096
	R3 (LX)	-.0121	p < 0.001	-.0191	-.0051
	R7 (MX)	-.0105	p < 0.001	-.0175	-.0034
	R9 (T)	-.0106	p < 0.001	-.0176	-.0036
	R4 (MX)	-.0015	.999	-.0085	.0055
	R6 (HX)	-.0001	1.000	-.0071	.0070
R9 (T)	R1 (LX)	-.0055	.274	-.0125	.0016
	R8 (HX)	-.0059	.180	-.0129	.0011
	R2 (MX)	-.0061	.155	-.0131	.0010
	R3 (LX)	-.0015	.999	-.0085	.0055
	R7 (MX)	.0001	1.000	-.0069	.0072
	R5 (HX)	.0106	p < 0.001	.0036	.0176
	R4 (MX)	.0091	.002	.0021	.0161
	R6 (HX)	.0105	p < 0.001	.0035	.0175
R4 (MX)	R1 (LX)	-.0145	p < 0.001	-.0216	-.0075
	R8 (HX)	-.0150	p < 0.001	-.0220	-.0080
	R2 (MX)	-.0151	p < 0.001	-.0222	-.0081
	R3 (LX)	-.0106	p < 0.001	-.0176	-.0036
	R7 (MX)	-.0090	.003	-.0160	-.0019
	R5 (HX)	.0015	.999	-.0055	.0085
	R9 (T)	-.0091	.002	-.0161	-.0021
	R6 (HX)	.0014	.999	-.0056	.0084
R6 (HX)	R1 (LX)	-.0160	p < 0.001	-.0230	-.0090
	R8 (HX)	-.0164	p < 0.001	-.0234	-.0094
	R2 (MX)	-.0166	p < 0.001	-.0236	-.0095
	R3 (LX)	-.0120	p < 0.001	-.0190	-.0050
	R7 (MX)	-.0104	p < 0.001	-.0174	-.0034
	R5 (HX)	.0001	1.000	-.0070	.0071
	R9 (T)	-.0105	p < 0.001	-.0175	-.0035
	R4 (MX)	-.0014	.999	-.0084	.0056

**Table 16:** Results from a two-way ANOVA of the Semilandmark dataset.

Source	Type III Sum of Squares	df	Mean Square	F	p-value
Corrected Model	.143	35	.004	11.343	p<0.001
Intercept	.788	1	.788	2190.950	p<0.001
scanner	.004	3	.001	3.675	.013
user	.103	8	.013	35.776	p<0.001
scanner user	.036	24	.001	4.157	p<0.001
Error	.117	324	.000		
Total	1.048	360			
Corrected Total	.259	359			

**Table 17:** Tukey’s post hoc pairwise comparisons of scanning types for the Semilandmark dataset.

(I) scanner	(J) scanner	Mean Difference (I-J)	p-value	95% Confidence Interval	
				Lower Bound	Upper Bound
BR	CT	.0006	.997	-.0067	.0079
	M	-.0021	.875	-.0094	.0052
	NE	.0068	.079	-.0005	.0141
CT	BR	-.0006	.997	-.0079	.0067
	M	-.0027	.767	-.0100	.0046
	NE	.0062	.129	-.0011	.0135
M	BR	.0021	.875	-.0052	.0094
	CT	.0027	.767	-.0046	.0100
	NE	.0089	.009	.0016	.0162
NE	BR	-.0068	.079	-.0141	.0005
	CT	-.0062	.129	-.0135	.0011
	M	-.0089	.009	-.0162	-.0016

**Table 18:** Tukey’s post hoc pairwise comparisons of users for the Semilandmark dataset.

(I) user	(J) user	Mean Difference (I-J)	p-value	95% Confidence Interval	
				Lower Bound	Upper Bound
R1 (LX)	R8 (HX)	.0361	p<0.001	.0229	.0494
	R2 (MX)	.0127	.070	-.0005	.0260
	R3 (LX)	-.0227	p<0.001	-.0360	-.0095
	R7 (MX)	.0237	p<0.001	.0105	.0370
	R5 (HX)	.0236	p<0.001	.0104	.0369
	R9 (T)	.0119	.119	-.0014	.0251
	R4 (MX)	.0301	p<0.001	.0169	.0433
	R6 (HX)	.0245	p<0.001	.0113	.0377
R8 (HX)	R1 (LX)	-.0361	p<0.001	-.0494	-.0229
	R2 (MX)	-.0234	p<0.001	-.0366	-.0101
	R3 (LX)	-.0588	p<0.001	-.0721	-.0456
	R7 (MX)	-.0124	.088	-.0256	.0009
	R5 (HX)	-.0125	.082	-.0257	.0007
	R9 (T)	-.0242	p<0.001	-.0375	-.0110
	R4 (MX)	-.0060	.891	-.0193	.0072
	R6 (HX)	-.0116	.140	-.0249	.0016
R2 (MX)	R1 (LX)	-.0127	.070	-.0260	.0005
	R8 (HX)	.0234	p<0.001	.0101	.0366
	R3 (LX)	-.0355	p<0.001	-.0487	-.0222
	R7 (MX)	.0110	.194	-.0022	.0242
	R5 (HX)	.0109	.206	-.0024	.0241
	R9 (T)	-.0009	1.000	-.0141	.0124
	R4 (MX)	.0174	.002	.0041	.0306
	R6 (HX)	.0118	.127	-.0015	.0250
R3 (LX)	R1 (LX)	.0227	p<0.001	.0095	.0360
	R8 (HX)	.0588	p<0.001	.0456	.0721
	R2 (MX)	.0355	p<0.001	.0222	.0487
	R7 (MX)	.0465	p<0.001	.0332	.0597
	R5 (HX)	.0463	p<0.001	.0331	.0596
	R9 (T)	.0346	p<0.001	.0214	.0479
	R4 (MX)	.0528	p<0.001	.0396	.0661
	R6 (HX)	.0472	p<0.001	.0340	.0605
R7 (MX)	R1 (LX)	-.0237	p<0.001	-.0370	-.0105
	R8 (HX)	.0124	.088	-.0009	.0256
	R2 (MX)	-.0110	.194	-.0242	.0022
	R3 (LX)	-.0465	p<0.001	-.0597	-.0332
	R5 (HX)	-.0001	1.000	-.0134	.0131
	R9 (T)	-.0119	.121	-.0251	.0014
	R4 (MX)	.0064	.855	-.0069	.0196
	R6 (HX)	.0008	1.000	-.0125	.0140
R5 (HX)	R1 (LX)	-.0236	p<0.001	-.0369	-.0104
	R8 (HX)	.0125	.082	-.0007	.0257
	R2 (MX)	-.0109	.206	-.0241	.0024
	R3 (LX)	-.0463	p<0.001	-.0596	-.0331
	R7 (MX)	.0001	1.000	-.0131	.0134
	R9 (T)	-.0117	.130	-.0250	.0015
	R4 (MX)	.0065	.841	-.0068	.0197
	R6 (HX)	.0009	1.000	-.0124	.0141
R9 (T)	R1 (LX)	-.0119	.119	-.0251	.0014
	R8 (HX)	.0242	p<0.001	.0110	.0375
	R2 (MX)	.0009	1.000	-.0124	.0141
	R3 (LX)	-.0346	.000	-.0479	-.0214
	R7 (MX)	.0119	.121	-.0014	.0251
	R5 (HX)	.0117	.130	-.0015	.0250
	R4 (MX)	.0182	.001	.0050	.0315
	R6 (HX)	.0126	.076	-.0006	.0259
R4 (MX)	R1 (LX)	-.0301	p<0.001	-.0433	-.0169
	R8 (HX)	.0060	.891	-.0072	.0193
	R2 (MX)	-.0174	.002	-.0306	-.0041
	R3 (LX)	-.0528	p<0.001	-.0661	-.0396
	R7 (MX)	-.0064	.855	-.0196	.0069
	R5 (HX)	-.0065	.841	-.0197	.0068
	R9 (T)	-.0182	.001	-.0315	-.0050
	R6 (HX)	-.0056	.925	-.0188	.0077
R6 (HX)	R1 (LX)	-.0245	p<0.001	-.0377	-.0113
	R8 (HX)	.0116	.140	-.0016	.0249
	R2 (MX)	-.0118	.127	-.0250	.0015
	R3 (LX)	-.0472	p<0.001	-.0605	-.0340
	R7 (MX)	-.0008	1.000	-.0140	.0125
	R5 (HX)	-.0009	1.000	-.0141	.0124
	R9 (T)	-.0126	.076	-.0259	.0006
	R4 (MX)	.0056	.925	-.0077	.0188

**Fig 6:**
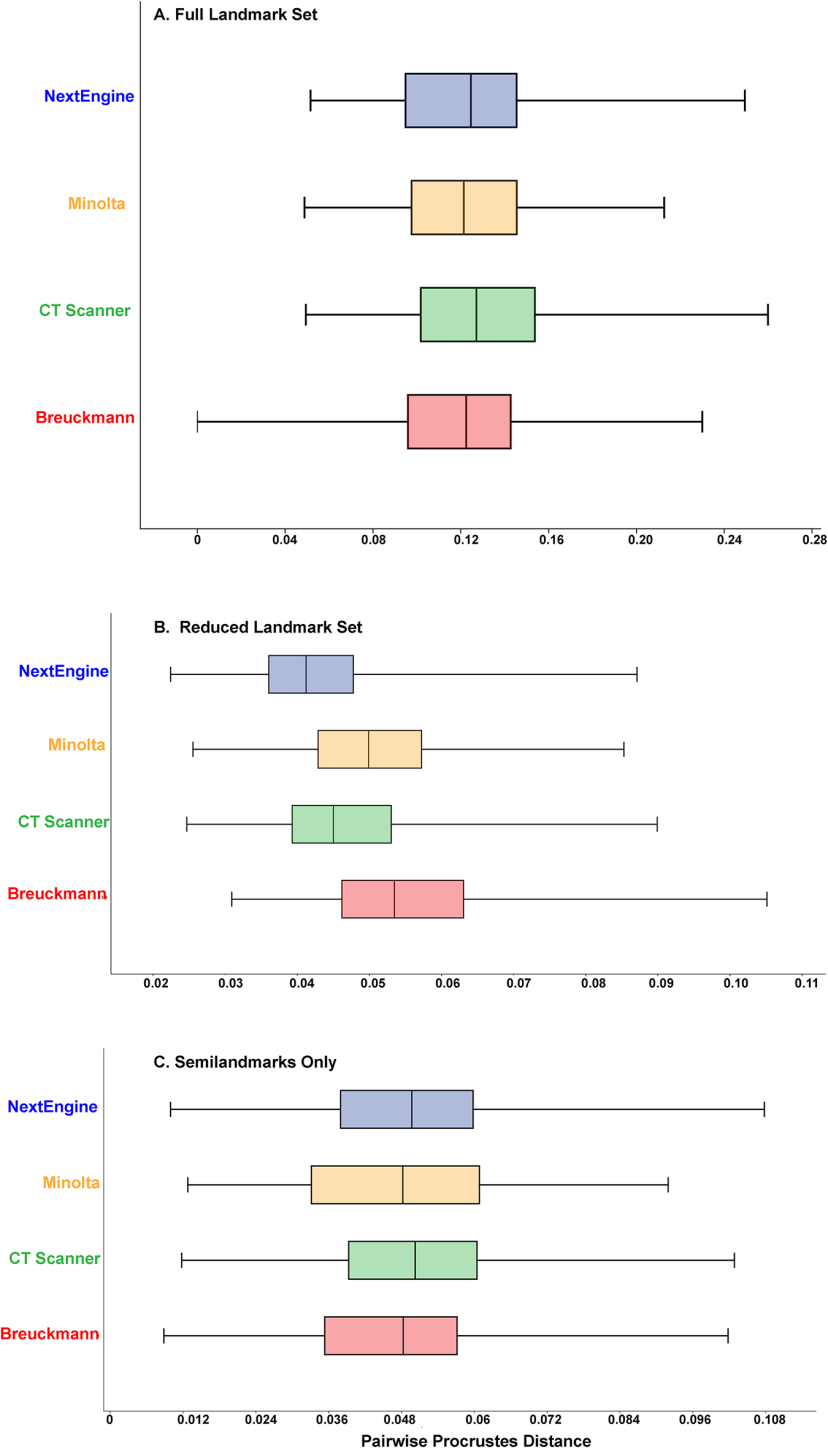
Boxplot of the distribution of pairwise Procrustes distances between different users for each scanner and landmark configuration. (A) Full landmark set; (B) Reduced landmark set; (C) Semilandmark set.

Fig 6 illustrates the distribution of pairwise Procrustes distances among different users–the equivalent in this case to interobserver error—among scan types for each of the three configurations. ANOVAs show no significant differences in the distribution of interobserver error among the four scanners tested for any of the three landmark configurations.

### Effects of user experience on error

**Fig 7:**
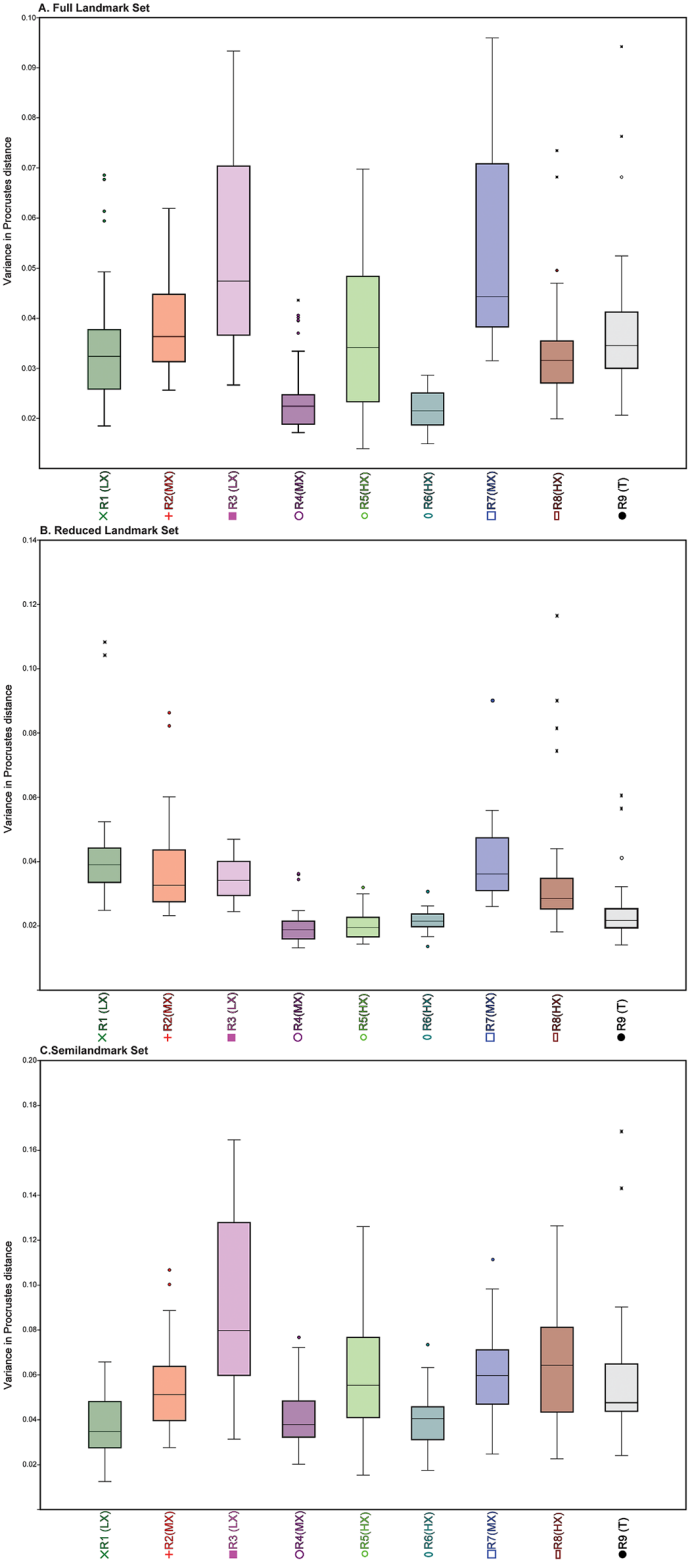
Boxplot illustrating the range of intraobserver error for each researcher for all forty trials. (A) Full landmark set; (B) Reduced landmark set; (C) Semilandmark set.

Fig 7 and Table 6 illustrate the variance in pairwise Procrustes distances for each researcher by landmark configuration. In most cases, researcher experience strongly correlated with levels of variance; less experienced researchers had higher levels of variance (e.g., R2 (MX) and R3 (LX); Table 18) and more experienced researchers had lower levels (e.g., R5 (HX), R6 (HX) and R9 (T)). Interestingly, Researcher 4 also had low levels of variance overall despite having equivalent experience as R2 (MX) and R7 (MX), so factors other than experience can play a role in obtaining a higher level of precision. R1 (LX) had the least experience and had relatively high levels of variance except in semilandmark placement where the researcher had lower variance than the others. R8 (HX) has intermediate levels of variance, sometimes being quite low and other times being quite high. For instance, R8 (HX) had lower levels of variance for the Reduced landmark set, except for the NextEngine trials, but much higher levels of variance for the curve set, regardless of scan type (Fig 7).

To examine rates of intraobserver error, we used ANOVA analyses with Tukey’s post hoc pairwise comparisons. For the Full landmark configuration, R4 (MX) and R6 (HX) were not significantly different from each other in landmark placement, but both had significantly lower rates of intraobserver error than other researchers. R3 (LX) and R7 (MX) were also not significantly different from each other, but both had significantly higher rates of intraobserver error. In the Reduced landmark set, there were no significant differences between R4 (MX), R5 (HX), R6 (HX) and R9 (T), but all four had significantly lower intraobserver error rates than the rest of the researchers. For the Semilandmark set, R3 (LX) had significantly higher values than all other researchers. R1 (LX), R3 (LX) and R6 (HX) were all not significantly different from each other, and all had significantly lower intraobserver rates than R5 (HX), R7 (MX), and R8 (HX) (in addition to R3 (LX)). The other researchers had mid-range values and did not form any cohesive groups.

Variability on the level of the individual can be seen in the results of the percent variance on the first three axes of our principal components analyses for all scans (Table 19). In most cases, the percent variance on the first three axes was relatively uniform; however, both R5 (HX) and R7 (MX) showed a higher proportion of variance on the first PC axis. Landmarks 1, 2, 13, 22, 23, 32 and 33 commonly had the greatest variance, and landmarks 3 and 31 the least; however, there was no consistent pattern as to the direction in which these landmarks varied for each user and no correlation between variance in location of these landmarks and scan type, suggesting these differences were stochastic in nature. In addition, no consistent pattern emerged when visualizing which landmarks contributed most to differences in landmark positions among scanners for each user along the first three principal axes.

**Table 19:** Percent of variance on the first three axes from principal component analyses by user for each landmark set combining all scan types and replicates (n = 40 combined scans per user).

Researcher	Full Landmark	Reduced Landmark	Semilandmark Only
1 (LX)	PC 1: 26.4%	PC 1: 29.9%	PC 1: 49.7%
	PC 2: 15.2%	PC 2: 16.9%	PC 2: 22.2%
	PC 3: 12.1%	PC 3: 14.2%	PC 3: 10.6%
2 (MX)	PC 1: 33.9%	PC 1: 31.7%	PC 1: 52.8%
	PC 2: 10.4%	PC 2: 11.7%	PC 2: 13.4%
	PC 3: 9.9%	PC 3: 10.7%	PC 3: 8%
3 (LX)	PC 1: 39.1%	PC 1: 16.4%	PC 1: 46.5%
	PC 2: 19.9%	PC 2: 14.1%	PC 2: 20.4%
	PC 3: 9.0%	PC 3: 9.4%	PC 3: 11.6%
4 (MX)	PC 1: 33.4%	PC 1: 35.0%	PC 1: 54.0%
	PC 2: 25.6%	PC 2: 14.1%	PC 2: 22.4%
	PC 3: 8.8%	PC 3: 7.9%	PC 3: 8.9%
5 (HX)	PC 1: 87.6%	PC 1: 37.5%	PC 1: 92.7%
	PC 2: 1.9%	PC 2: 12.8%	PC 2: 1.6%
	PC 3: 1.4%	PC 3: 6.1%	PC 3: 1.2%
6 (HX)	PC 1: 28.5%	PC 1: 28.5%	PC 1: 34.6%
	PC 2: 14.7%	PC 2: 14.7%	PC 2: 17.4%
	PC 3: 11.1%	PC 3: 11.1%	PC 3: 8.0%
7 (MX)	PC 1: 54.7%	PC 1: 78.3%	PC 1: 37.9%
	PC 2: 17.2%	PC 2: 7.2%	PC 2: 22.6%
	PC 3: 8.2%	PC 3: 2.9%	PC 3: 15.6%
8 (HX)	PC 1: 20.6%	PC 1: 30.3%	PC 1: 33.1%
	PC 2: 16.7%	PC 2: 20.3%	PC 2: 21.5%
	PC 3: 11.6%	PC 3: 11.0%	PC 3: 10.6%
9 (T)	PC 1: 25.2%	PC 1: 35.5%	PC 1: 35.8%
	PC 2: 21.9%	PC 2: 15.8%	PC 2: 24.7%
	PC 3: 13.5%	PC 3: 8.3%	PC 3: 10.2%

### Effects of in-person training on error

**Fig 8:**
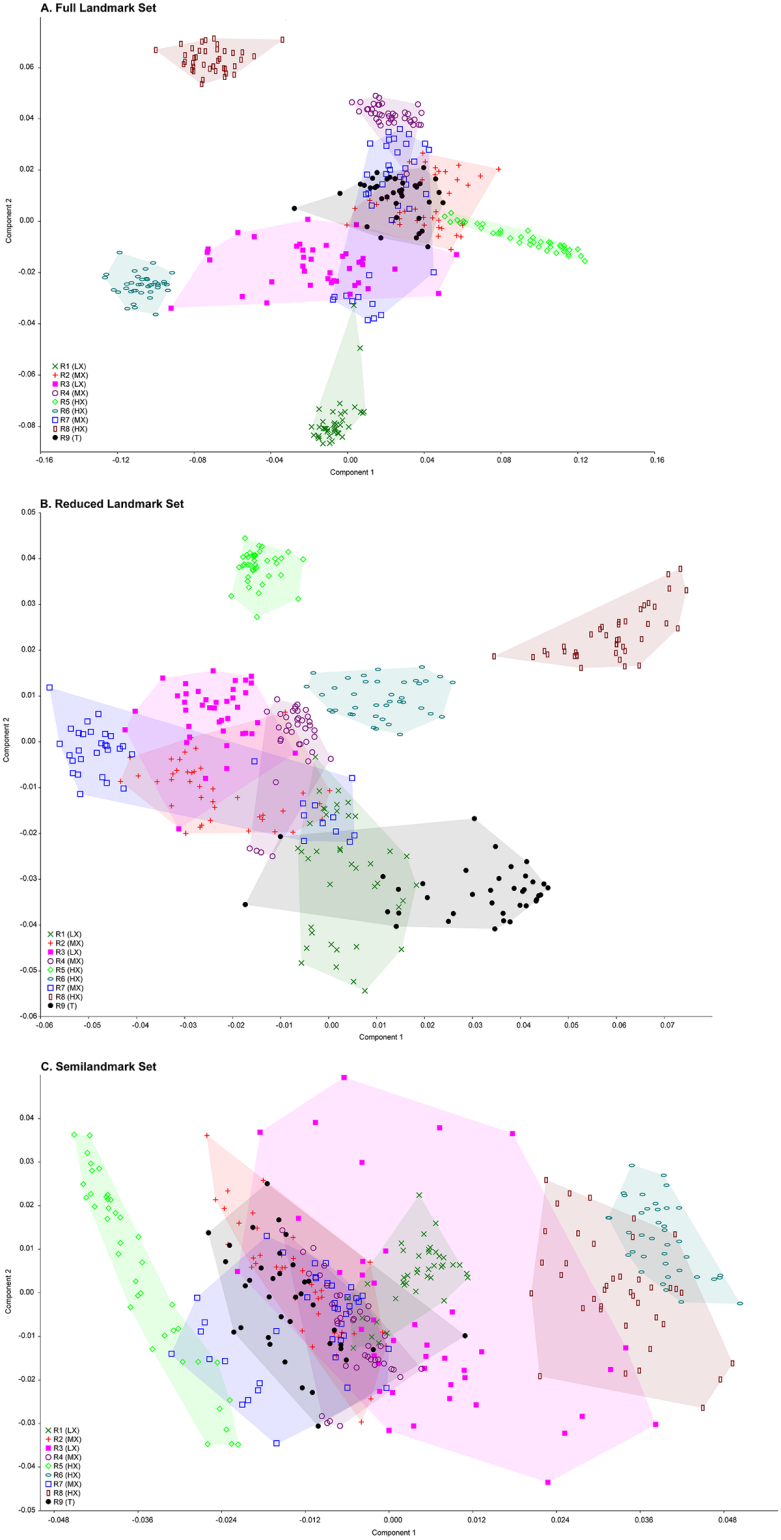
PCA plots of all trials from all users. (A) Full landmark set; (B) Reduced landmark set; (C) Semilandmark set.

Fig 8 depicts a PCA plot of all the iterations of the full landmark set for all researchers. R2 (MX), R3 (LX), R4 (MX), and R7 (MX) all received individual training from R9 (T) and broadly overlap in their landmark placements towards the center of the PC axes for the full landmark set (Fig 8(A). R1 (LX) also received in-person training, but falls farther away from R9 (T) on PC 2. R6 (HX) has similar values to the training group on PC 2 but falls more towards the negative axis of PC 1. R8 (HX) is different from the training group on both PC 1 and PC 2. For the Reduced landmark set (Fig 8(B), there is almost complete overlap between R2 (MX), R3 (LX), R4 (MX) and R7 (MX), all of whom had in-person training. R1 (LX) partially overlaps with this group. Two of the trials from R9 (T) fall with this group, but most of R9 (T)’s trials are separated from the training group on both PC 1 and PC 2. R5 (HX) and R6 (HX) fall with the training group on PC 1 but not PC 2. Again, R8 (HX) is farther away on both axes. In the Semilandmark only set (Fig 8(C), PC 1 accounts for the differences among researchers while PC 2 represents variation related to intraobserver error. Most of the researchers with in-person training fall with R9 (T) on this axis. R6 (HX) and R8 (HX) are most distant from this cluster at the positive end of PC 1, while R5 (HX) with just in-person clarification of details falls on the negative end of this axis.

**Fig 9:**
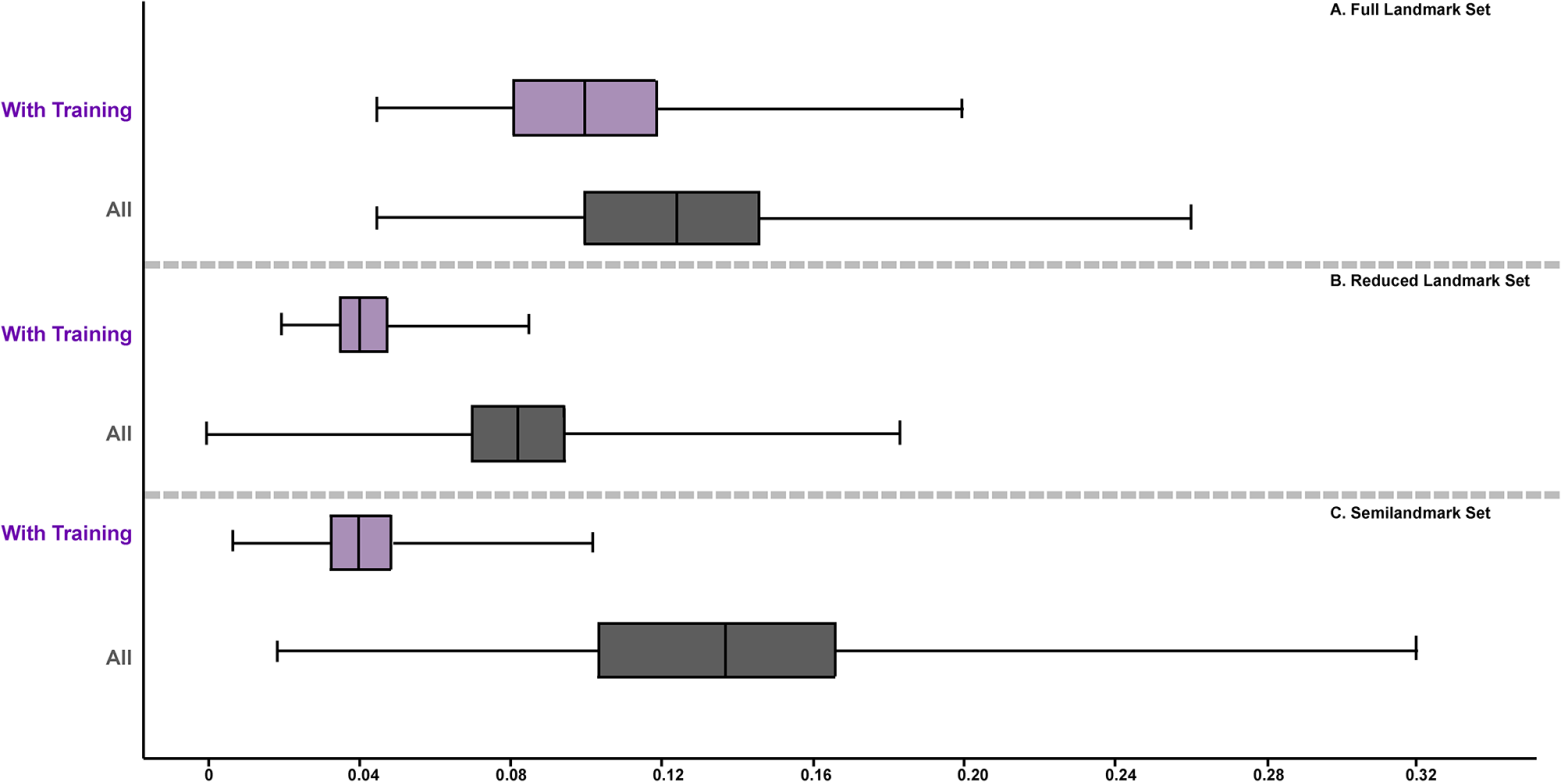
Boxplots illustrating the change in interobserver error when those without in-person training were removed. (A) Full landmark set; (B) Reduced landmark set; (C) Semilandmark set.

**Fig 10:**
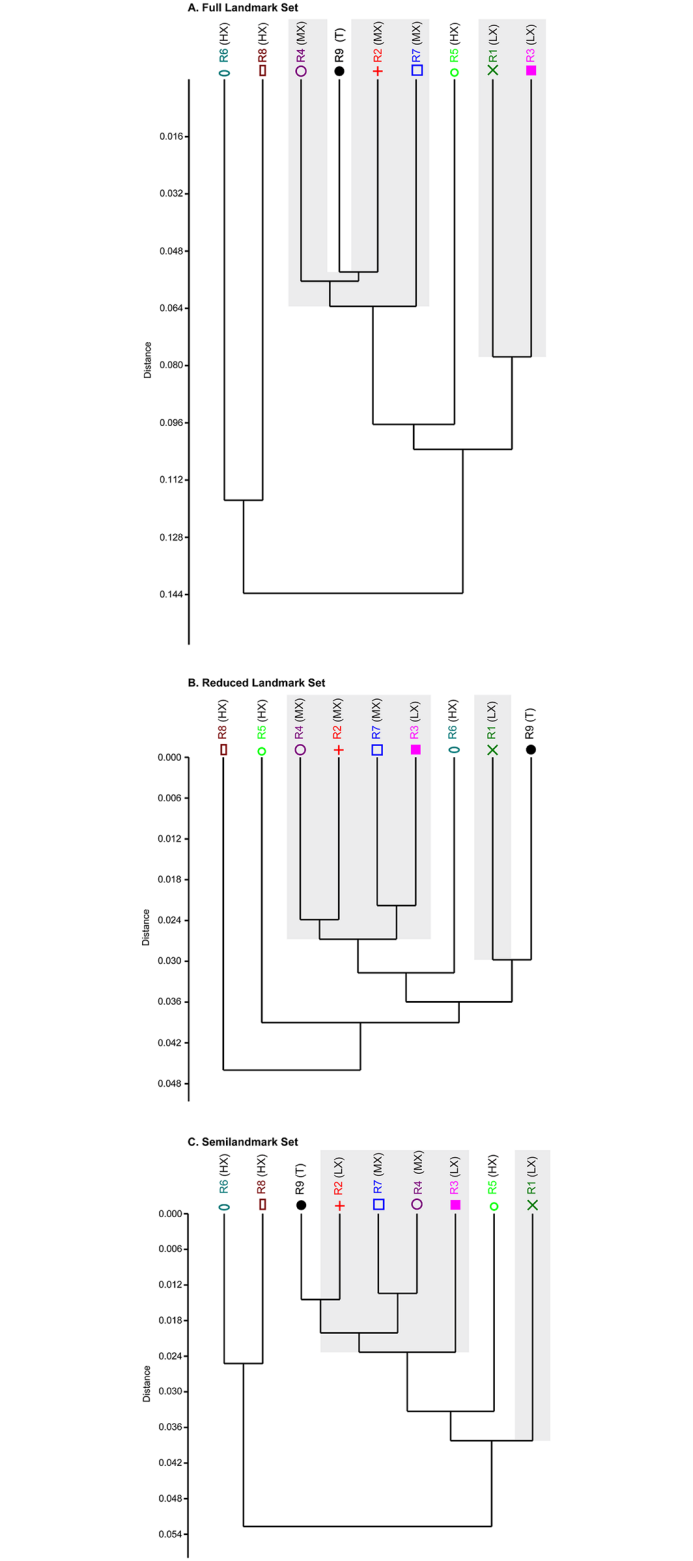
UPGMA dendrograms illustrating how different researchers cluster. (A) Full landmark set; (B) Reduced landmark set; (C) Semilandmark set. Gray areas represent individuals that received in-person training by R9.

Removing users who had no in-person training from R9 (T) did improve average interobserver error for two of the datasets. Average interobserver error was improved for the Full landmark (0.12 to 0.10) and Semilandmark only sets (0.14 to 0.11) but not for the Reduced landmark set (0.08) (Fig 9). A dendrogram (Fig 10) based on each landmark set of all trial iterations indicates that most users who received in-person training from R9 (T) clustered with R9 (T) for the Full and Semilandmark only datasets. In the Full dataset (Fig 10(A), two experienced users with no input from R9 (T) (i.e. R6 (HX), R8 (HX)) form an outgroup cluster to the remaining researchers that did receive training, excepting R5 (HX), who clusters as a sister group of R9 (T) plus trainees to the exclusion of R1 (LX) and R3 (LX), who also received in person training from R9 (T). For the Reduced landmark set, four of five users who received training (R2 (MX), R3 (LX), R4 (MX), and R7 (MX)) from R9 (T) form a cluster with each other, and R9 (T) forms a group with R1 (LX) (trainee) in a separate cluster. R5 (HX) and R8 (HX) (who received no in-person training) fall outside the trainee group, although R6 (HX) falls as sister to the main trainee cluster, suggesting some similarity in marking with the Reduced landmark set. Using the Semilandmark only set, the dendrogram clusters all trainees except for R1 (LX) close to the trainer R9 (T), although R5 (HX) (non-trainee) splits the two groups.

### Interobserver error vs. shape variance among multiple specimens

**Fig 11:**
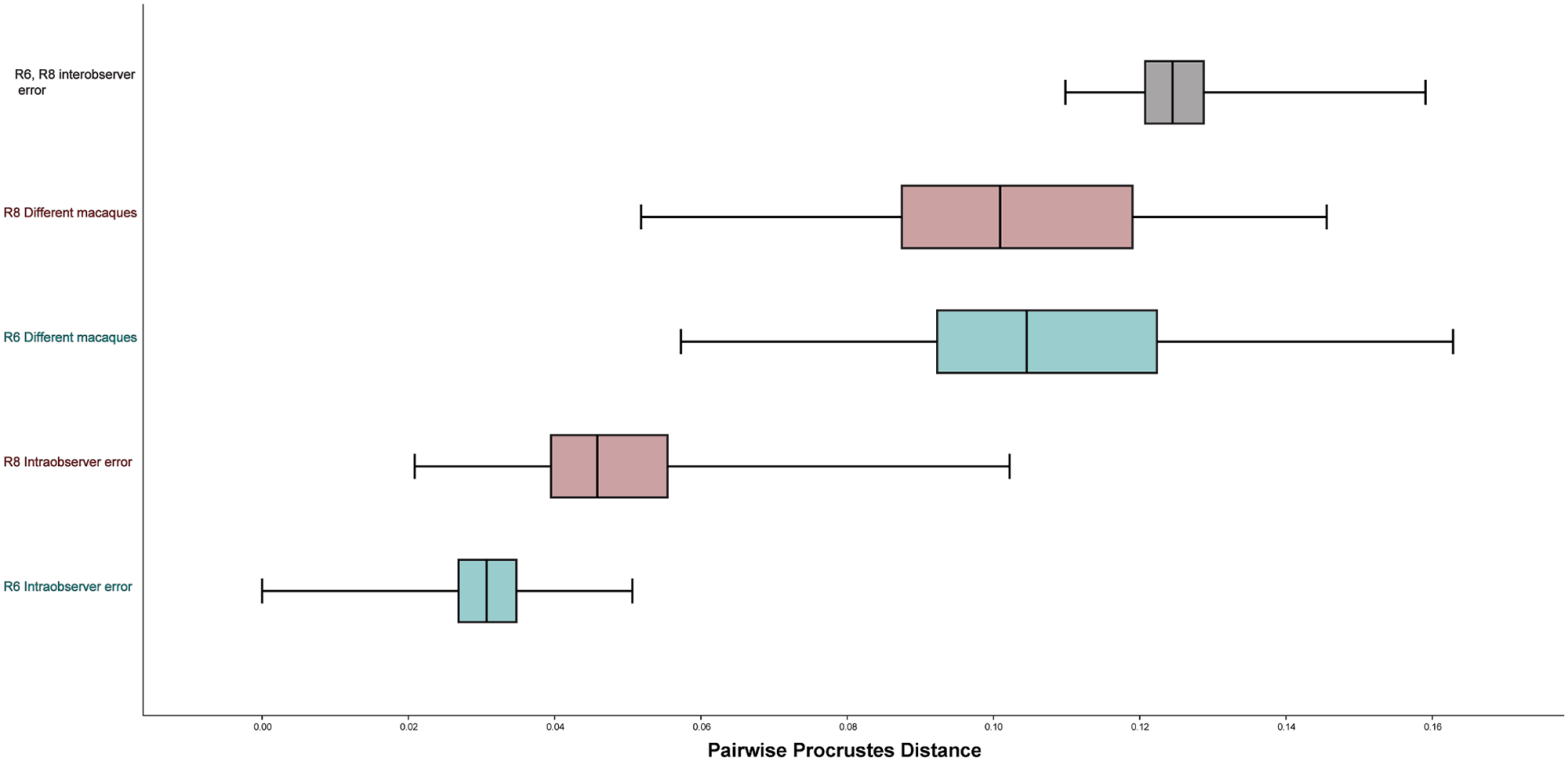
Boxplots comparing inter- and intraobserver for Researchers 6 and 8 relative to the variation found among difference species of *Macaca*. These data are from the full landmark set.

Fig 11 illustrates a comparison between the range of inter- and intraobserver error for two researchers (R6 (HX) and R8 (HX)) compared to the range of shape difference among the crania of ten different macaques from seven different species. For the Full data set, average interobserver error was greater than the differences between different macaques. However, for both the Reduced and the Semilandmark only set, the average difference between different macaques was greater than interobserver error (Table 20). That said, in all three landmark configurations the range of pairwise Procrustes distances representing interobserver error overlapped substantially with the range of pairwise Procrustes distances between the different macaque crania. In addition, the distribution of pairwise Procrustes distances representing intraobserver error also overlapped with the distribution of pairwise Procrustes distances between different macaques for the Semilandmark only set for both researchers. Intraobserver error for R8 (HX) also slightly overlapped the differences among macaques for the Full and Reduced landmark configurations; intraobserver error for R6 (HX) did not overlap the distribution of pairwise Procrustes distances for different macaques at all for these two datasets (Fig 11).

**Table 20:** Average pairwise Procrustes distance between landmarked trials by the same user (intraobserver error), landmarked trials between two different users (interobserver error) and between different macaques.

	Full	Full with Sliding	Reduced	Semilandmark	Semilandmark with Sliding
R6 (HX) intraobserver error	0.03	0.02	0.03	0.05	0.03
R8 (HX) intraobserver error	0.05	0.04	0.05	0.09	0.07
R6 (HX) different macaques	0.11	0.10	0.13	0.13	0.10
R8 (HX) different macaques	0.10	0.09	0.12	0.12	0.10
interobserver error	0.13	0.11	0.08	0.10	0.09

**Fig 12:**
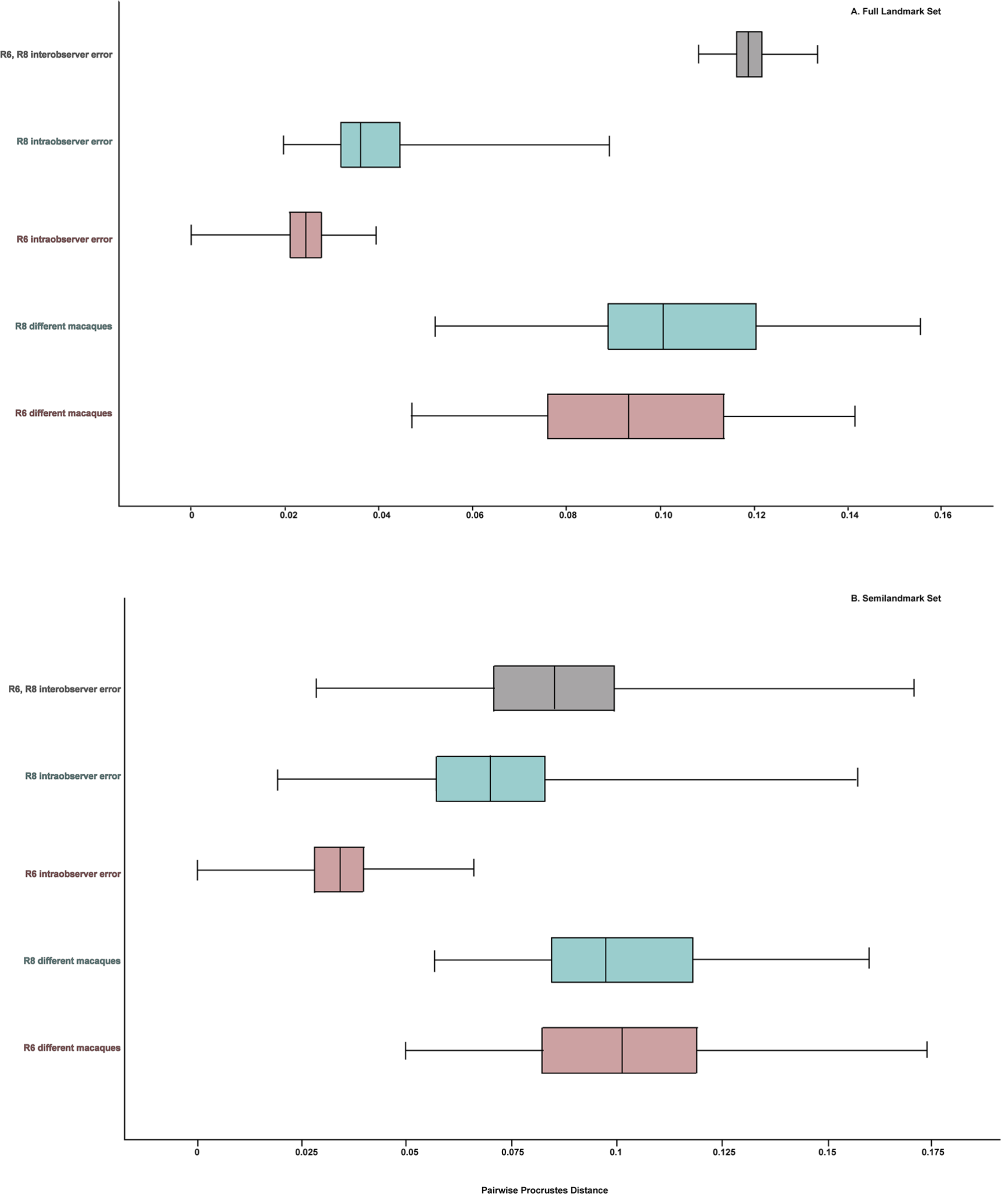
Boxplots comparing inter- and intraobserver error Researchers 6 and 8 to the variation in different species of *Macaca* for the Full and Semilandmark only configurations after semilandmark sliding. (A) Full landmarkset; (B) Semilandmark set. Note the low amount of pooled intraobserver error relative to the large amount of interobserver error between the researchers and relative to the amount of variation in different species of macaques for both landmark configurations.

In both landmark sets, sliding semilandmarks reduced intraobserver error as well as the differences among the different macaques (Fig 12). Sliding the semilandmarks seemed to have the most obvious impact on intraobserver error vs. the differences among the macaque crania for each of the users separately. For instance, for R6 (HX), after semilandmark sliding there was almost no overlap between the range of Procrustes distances among the repetitions and among the different macaques for the Semilandmark only set. However, sliding the semilandmarks did not have an appreciable effect on lowering the interobserver error; in fact, for the Full configuration, mean interobserver error increased as compared to no semilandmark sliding (Table 20). In both landmark sets mean interobserver error is close to the mean Procrustes distance between different macaque crania.

## Discussion

Here, we present results of an error study comparing compatibility of scan types–which vary by instruments and scan acquisition protocol–on user-gathered landmark data to determine the extent to which error within and among individuals can influence the outcome of a geometric morphometric study. We evaluated these factors to determine whether or not it is sound practice to combine data collected from multiple scanners and/or by multiple individuals. The trend of data sharing and increased availability of both scan and landmark data present challenging questions about both compatibility of datasets and repeatability of landmarks given the potential that a researcher may use multiple scanners for a project and involve multiple co-workers in data collection. Overall, we observed three major trends in our data and offer suggestions on how to mitigate the problems arising from such trends:

### (1) Error rates appear to remain consistent among and within users regardless of overall scan quality or type

Based purely on visual assessment, distinctly different digital models result from all the surface scanners and CT scanner tested here (see Figs 2 and 3), each with clearly observable differences in surface texture and resolution. For example, the two laser surface scanners do not capture the morphology of the teeth well, most likely due to the refractive properties of enamel and/or lower inherent resolving power. Similarly, complex structures like the basicranium are not captured as well by the laser surface scanners when compared to the white light scanner and the CT scanner.

When all researchers are considered together, no distinct pattern emerges to designate a clearly superior scan type to reduce landmark error. There were significant differences among scan types at the level of an individual researcher, but there was no pattern as to which scan types were significantly different from one another, or which scan types resulted in the lowest levels of intraobserver error. In other words, any statistically significant differences in any researcher's trials do not reflect a broad pattern, but rather more likely reflect individual inconsistencies in landmarking. Thus, despite the visible differences, scan model was not found to significantly influence most researchers' abilities to place landmarks and did not affect overall intra- and interobserver error rates (see Table 18 and Figs 4 and 5). This finding is consistent with that of Terhune and Robinson [17] although not with Fruciano and colleagues [18]. That said, Fruciano and colleagues [18] used a different set of scan types than this study or Terhune and Robinson [17]. Additionally, Fruciano and colleauges [18] reduced the complexity of their higher resolution scan (taken by a Solutionix Rexcan CS+ scanner) to match the triangle count of the Nextengine scanner, which is a protocol that neither Terhune and Robinson [17] or we report as part of our model construction protocol. This difference in post-processing may account for some of the reported differences. Finally, we did find some significant differences among surface scanners in this study, though the effect size was similar to (or smaller than) intraobserver error. Similar metrics are not reported in Fruciano et al. [18], so it is difficult to determine whether their results match this study in term of effect size. However, differences in initial design are apparent, and have undoubtedly influenced the results of our separate studies. As Fruciano et al. [18] differed from our study in several ways (e.g., smaller number of participants, narrow range of participant experience, exclusive use of Type I landmarks), we expect that the discrepancies with our results are likely the downstream effects of differences in basic design features.

In this study, as higher scan quality did not consistently reduce error and lower scan quality did not increase error, we believe that scanner type may reflect a case of diminishing returns, whereby even the lowest quality modern scanner will maintain a resolution sufficient for accurate and precise landmarking, while higher resolution scanners may not improve on this model resolution drastically enough to influence results. On the other hand, such differences in resolution may impact the clarity of the scan when used in observations of morphology, e.g., for scoring characters to be used in a cladistic analysis, a question not addressed here.

### (2) Users with more osteology and 3DGM experience generally had less intraobserver error, but experience with osteology or morphometrics did not improve interobserver error

Researchers with little experience were less likely to be consistent within their own scan iterations, but researchers with extensive levels of experience did not necessarily agree on point collection protocol, and therefore have similar levels of interobserver variance as the inexperienced users. For example, R1 (LX), R4 (MX), R6 (HX), and R9 (T) maintained high precision throughout their trials but disagreed on what constituted accurate landmark placement. The data clusters for R1 (LX) and R4 (MX) occupy a similar morphospace on PC 1, but are on opposite ends of PC 2, a trend that R6 (HX) and R8 (HX) also share, although both R6 (HX) and R8 (HX) are shifted to the positive end of PC 1 relative to R1 (LX) and R4 (MX).

However, if broken into two groups—those that received in-person training in point collection from R9 (T) and those that did not—individuals who received training in landmark placement had lower average interobserver error rates when compared with each other than those that did not for the landmark configurations including semilandmarks. This trend persists despite the fact that the group that received training had relatively greater intraobserver error and less overall experience. These results suggest that in-person training for a particular landmark collection protocol could be critical in mitigating the effects of interobserver error, but we acknowledge that this is an impractical step for researchers interested in sharing their landmark data via digital media. We therefore suggest planning ahead if intending to combine landmark data from multiple researchers by providing at the start of a project extremely detailed data collection guides where relevant with photographs and clear written descriptions, i.e., a higher level of training than was provided by R9 (T) in this study, especially for datasets that include semilandmarks. Additionally, a pre-landmarked “Atlas” specimen provided by the dataset’s originator may prove useful as a template exemplar for less experienced users or for complex point arrangements, although to what extent this may improve rates of interobserver error remains to be tested. We recommend that any study using landmark data from multiple researchers must be carefully designed with these potential sources of error in mind from the start; it is not advisable to simply mine online databases, or make requests of colleagues for previously collected landmark data to combine into one master data set. Detailed guides and initial supervision are critical for any study combining data from multiple sources.

### (3) Interobserver error was consistently higher than all other potential error types observed among researchers in this study

Our results suggest that interobserver error is of much greater concern than intraobserver error for different scan types or scan iterations. The average amount of variance between users landmarking a single cranium was roughly equivalent to, and in some cases greater than, the average amount of shape variation found among single cranial representatives from ten different macaques (Fig 12). R6 (HX) and R8 (HX) were chosen among the HX researchers to complete this trial; it is possible that interobserver error would have been substantially lower had different researchers completed this set of trials. Sliding semilandmarks improved intraobserver error in these trials, but actually increased interobserver error, so we do not recommend using semilandmark sliding as a strategy to decrease interobserver error. This finding impels caution in combining scan-based 3DGM datasets without first conducting numerous error tests to minimize variance. The potential for noise to mask real biological differences is a genuine concern for many researchers, and combining data collected by multiple individuals may in fact overwhelm any real signal in data.

## Conclusions

Overall, our results suggest that interobserver error is of much greater concern than intraobserver error for different scanners or scan iterations in 3DGM studies using landmarks collected on virtual specimens. The average amount of interobserver error on the same specimen was approximately equivalent to the average pairwise Procrustes differences among ten different macaques, suggesting that interobserver error may be mistaken for real biological differences where none actually exist if data collected by multiple users are combined in a study. As such, our results impel caution when attempting to combine landmark-based datasets from multiple individuals, and we suggest that multiple error studies be conducted within and among involved researchers to mitigate both intra- and interobserver error before data collection intended for publication is conducted. Our results also suggest that error rates can be reduced if researchers participating in a study receive specific, in-person instruction from one individual or agree via consensus on data collection protocols. Digital data sharing efforts in morphometrics should be approached with great caution unless the consistency of a landmarking protocol is carefully verified in this way. Moreover, as scanner type appears to have minimal influence on landmark variance, we encourage that scans, rather than landmarks, should be shared.
